# Systematic Analysis of Copy Number Variations in the Pathogenic Yeast Candida parapsilosis Identifies a Gene Amplification in *RTA3* That is Associated with Drug Resistance

**DOI:** 10.1128/mbio.01777-22

**Published:** 2022-09-19

**Authors:** Sean A. Bergin, Fang Zhao, Adam P. Ryan, Carolin A. Müller, Conrad A. Nieduszynski, Bing Zhai, Thierry Rolling, Tobias M. Hohl, Florent Morio, Jillian Scully, Kenneth H. Wolfe, Geraldine Butler

**Affiliations:** a School of Biomolecular and Biomedical Science, Conway Institute, University College Dublin, Belfield, Dublin, Ireland; b Sir William Dunn School of Pathology, University of Oxford, Oxford, United Kingdom; c Earlham Institute, Norwich, United Kingdom; d School of Biological Sciences, University of East Anglia, Norwich, United Kingdom; e Infectious Disease Service, Department of Medicine, Memorial Sloan Kettering Cancer Center, New York, New York, USA; f Immunology Program, Sloan Kettering Institute, Memorial Sloan Kettering Cancer Center, New York, New York, USA; g Laboratory of Quantitative Engineering Biology, Shenzhen Institute of Synthetic Biology, Shenzhen Institutes of Advanced Technology, Chinese Academy of Sciences, Shenzhen, China; h Department of Medicine, Weill Cornell Medical College, New York, New York, USA; i Nantes Université, CHU de Nantes, Cibles et Médicaments des Infections et de l'Immunité, IICiMed, Nantes, France; j School of Medicine, Conway Institute, University College Dublin, Belfield, Dublin, Ireland; Tel Aviv University

**Keywords:** *Candida*, copy number variation, genomics, drug resistance evolution

## Abstract

We analyzed the genomes of 170 C. parapsilosis isolates and identified multiple copy number variations (CNVs). We identified two genes, *RTA3* (*CPAR2_104610*) and *ARR3* (*CPAR2_601050*), each of which was the target of multiple independent amplification events. Phylogenetic analysis shows that most of these amplifications originated only once. For *ARR3*, which encodes a putative arsenate transporter, 8 distinct CNVs were identified, ranging in size from 2.3 kb to 10.5 kb with 3 to 23 copies. For *RTA3*, 16 distinct CNVs were identified, ranging in size from 0.3 kb to 4.5 kb with 2 to ~50 copies. One unusual amplification resulted in a DUP-TRP/INV-DUP structure similar to some human CNVs. *RTA3* encodes a putative phosphatidylcholine (PC) floppase which is known to regulate the inward translocation of PC in Candida albicans. We found that an increased copy number of *RTA3* correlated with resistance to miltefosine, an alkylphosphocholine drug that affects PC metabolism. Additionally, we conducted an adaptive laboratory evolution experiment in which two C. parapsilosis isolates were cultured in increasing concentrations of miltefosine. Two genes, *CPAR2_303950* and *CPAR2_102700*, coding for putative PC flippases homologous to S. cerevisiae *DNF1* gained homozygous protein-disrupting mutations in the evolved strains. Overall, our results show that C. parapsilosis can gain resistance to miltefosine, a drug that has recently been granted orphan drug designation approval by the United States Food and Drug Administration for the treatment of invasive candidiasis, through both CNVs or loss-of-function alleles in one of the flippase genes.

## INTRODUCTION

Copy number variations (CNVs), changes in the number of copies at a genomic location, are common in biological systems (1, 2). Many CNVs in human cells are associated with disease, particularly cancers (3). In addition, CNVs in yeasts are frequently identified in industrial isolates and during evolution experiments that examine adaptations to exogenous compounds or to limiting environmental conditions. One of the best-studied examples is the amplification of the *CUP1* metallothionein locus in Saccharomyces cerevisiae in response to the presence of toxic copper (4). An increased resistance is associated with the tandem amplification of the *CUP1* open reading frame (ORF) (5, 6). Similarly, limiting sulfate in S. cerevisiae induces the amplification of the *SUL1* gene, encoding a sulfate transporter, whereas limiting glucose and amino acids leads to the amplification of the glucose transporter *HXT6* and the amino acid transporter *GAP1*, respectively (1, 2, 7–9). Limiting glutamine also induces the amplification of the urea permease *DUR3* (2). Several different amplifications of *SUL1* and *GAP1* were observed in laboratory evolution experiments, differing in copy number and in the boundaries of the amplification units (2, 7). In addition, the exposure of the pathogenic yeast Candida albicans to antifungal drugs, such as azoles, is associated with multiple changes in the genome, including CNVs (10–12).

Multiple CNVs have been identified in natural isolates of many yeasts, including S. cerevisiae (13), C. albicans (14, 15), and Candida glabrata (16). Many affect genes in subtelomeric regions, which are known hot spots for variation (17, 18). Some CNVs in S. cerevisiae are lineage-specific, occurring particularly in industrial isolates, and are associated with specific phenotypes (19, 20). However, CNVs in natural isolates that occur outside subtelomeric regions and that differ significantly in copy number and in the size and organization of the amplification unit are rare and are surprisingly poorly studied. *CUP1* is an exception; the copy number has been shown to vary from 0 to ~80 with different endpoints in natural and industrial isolates of S. cerevisiae (21–23), though most studies are of amplifications induced in the laboratory by growth in high copper concentrations (24, 25).

Changes in copy number can result from several different mechanisms (26). Where an array already exists (i.e., where there are already at least two copies of a gene at one allele), it can be expanded by unequal crossing-over (nonallelic homologous recombination, NAHR). A misalignment of the alleles results in different numbers of copies following mitosis or meiosis. This may underlie some of the natural variation observed at the *CUP1* locus in S. cerevisiae (22). NAHR between inverted repeats can also result in dicentric chromosomes, which are resolved through several breakage-fusion-bridge cycles and result in CNVs (27). These have been observed in azole-resistant isolates of C. albicans (10). Many CNVs in C. albicans that are induced by exposure to drugs are adjacent to long inverted repeat sequences (10). Some are complex structures, consisting, for example, of a symmetrical “stair-step” amplification, with copy number changes occurring in two steps.

Many CNVs have been hypothesized to be caused by replication-mediated mechanisms. For example, the *CUP1* locus is adjacent to an origin of replication, and the induction of the expression of *CUP1* causes the stalling of the replication fork at this origin (25). The stalled replication fork is repaired by strand invasion in a mechanism similar to break induced replication (BIR) (28). Errors in BIR at repeat regions can result in copy number variation. Microhomology-mediated BIR (MMBIR) occurs where there are short (micro) regions of homology between the collapsed fork and other single-stranded DNA (29). MMBIR may result in expansion at the *DUR3* locus and in many of the *GAP1* expansions described in S. cerevisiae (2).

Some unusual amplifications may require a combination of mechanisms. These include a structure with a triplicated inverted central copy surrounded by duplicated regions (DUP-TRP/INV-DUP) seen in some human CNVs (30). A similar structure was observed in some amplifications of *SUL1* in S. cerevisiae (31). The ODIRA (Origin-dependent inverted-repeat amplification) model proposes that these amplifications occur at regions containing an origin of replication flanked by short, inverted repeats. Slippage at one fork could generate closed, circular, self-complementary extrachromosomal intermediates, which are subsequently integrated at the original site (31).

Pryszcz et al. (32) described several CNVs in four isolates of the human fungal pathogen Candida parapsilosis, including the amplification of *ARR3*, a putative arsenate transporter. In addition, in 2020, we described an amplification in 23 related C. parapsilosis isolates that resulted in a dramatically increased copy number (24 to 33×) of the *RTA3* gene (33). Here, we used genome sequencing to explore CNVs in 170 isolates of C. parapsilosis from different sources. We identified 8 different amplifications of *ARR3*, and 3 examples of large stair-step amplifications that are similar to those described in C. albicans (10). Notably, we found 16 distinct amplifications of the region surrounding *RTA3* that have unique endpoints, indicative of independent and parallel amplification events. We also found an in-frame fusion to a related neighboring gene, *RTA2*. We identify one CNV with a DUP-TRP/INV-DUP structure at *RTA3* in four isolates. Some allelic expansions of *RTA3* may occur through NAHR, but we found that many isolates have only one copy of *RTA3* at each allele, suggesting that amplification must also occur by other means.

The amplification of *RTA3* increases its transcription and probably its translation. Rta3 is a member of the Rta1/Rsb1-like family in S. cerevisiae, which encodes putative transporters with seven transmembrane (TM) domains. ScRsb1 controls the localization of sphingoid bases in S. cerevisiae, including phytosphingosine and dihydrosphingosine (34, 35). Rsb1 is localized to the membrane and is likely to act as a membrane transporter (a floppase) (36) or possibly as a regulator of transporters (37). In C. albicans, CaRta3 localizes to the plasma membrane; a fluorophore-labeled PC accumulates in the inner leaflet of the membrane in a *CaRTA3* deletion (38). Deleting *CaRTA3* also decreases resistance to the drug miltefosine (hexadecylphosphocholine), an alkylphosphocholine derivative that inhibits PC biosynthesis or localization (39). *CaRTA3* is therefore likely to encode a PC floppase (38).

We find that the copy number of *RTA3* correlates with resistance to miltefosine in C. parapsilosis. However, experimentally-induced adaptation in the presence of miltefosine did not induce the amplification of *RTA3* but instead resulted in the inactivation of two flippase genes. Therefore, we show that natural copy number variation in C. parapsilosis results in drug resistance but that miltefosine is unlikely to be the cause of selection for amplification. In addition, we show that miltefosine, which was granted orphan drug designation approval by the United States Food and Drug Administration (FDA) for the treatment of invasive candidiasis (https://www.accessdata.fda.gov/scripts/opdlisting/oopd/detailedIndex.cfm?cfgridkey=843921), is a poor treatment choice for C. parapsilosis because resistant isolates arise easily in the population.

## RESULTS

### Phylogeny and CNVs in 170 C. parapsilosis isolates.

**FIG 1:**
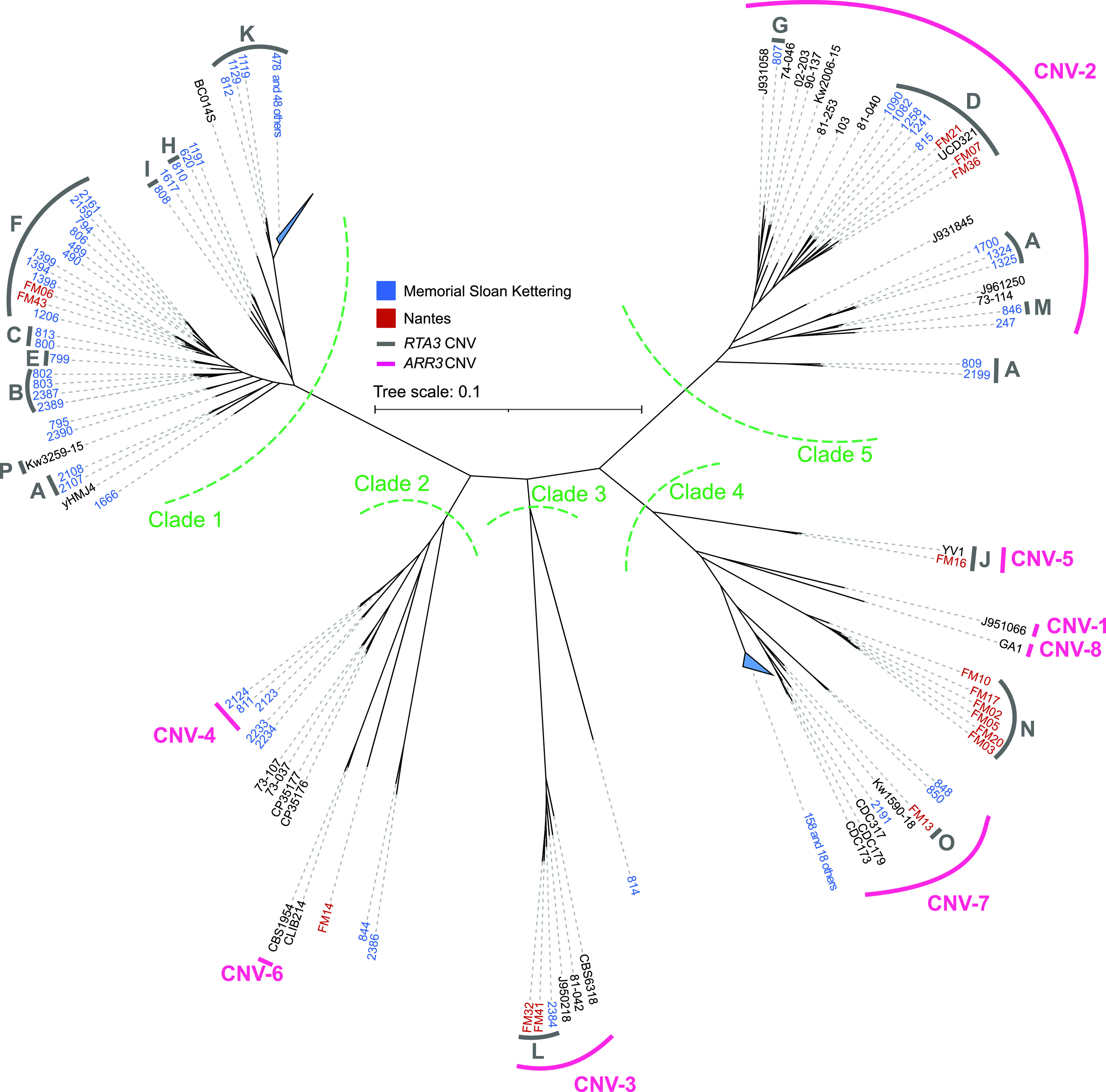
SNP-based unrooted phylogeny of 170 C. parapsilosis isolates. Isolates from Memorial Sloan Kettering Cancer Center (MSK) are named in blue (the prefix MSK is omitted from these strain names), and isolates from CHU de Nantes (FM) are named in red. Green dashed lines label each of five apparent clades. 49 similar isolates in Clade 1 and 19 similar isolates in Clade 4 were grouped and are shown as blue triangles. Isolates that harbor a CNV at *RTA3* are marked with a thick gray bar and a letter (from A to P) that corresponds to each of the 16 CNVs. The CNVs at *ARR3* are marked with a pink bar and a number (from CNV-1 to CNV-8). The phylogeny was constructed by calling SNPs for each sample using the GATK HaplotypeCaller tool (77). Filtered heterozygous sites were resolved using 1,000 iterations of random repeated haplotype sampling to provide haploid inputs for tree construction (71). The tree was then constructed using RAxML with the GTRGAMMA model of nucleotide substitution.

We explored the relationships of 170 Candida parapsilosis isolates using a genome-wide SNP alignment approach, generating the largest phylogeny of C. parapsilosis isolates to date (Fig. 1). Isolates were obtained from several locations, including some that were previously published and many that are sequenced and described here for the first time (Table S1). Most isolates originated from the Memorial Sloan Kettering Cancer Center in New York (MSK) and the Centre Hospitalier Universitaire de Nantes, France (FM) (Fig. 1).

10.1128/mbio.01777-22.2 TABLE S1 List of strains used. Download Table S1, DOCX file, 0.1 MB. Copyright © 2022 Bergin et al. 2022 Bergin et al. https://creativecommons.org/licenses/by/4.0/ This content is distributed under the terms of the Creative Commons Attribution 4.0 International license.

Isolates fall into five major clades, with almost half (84 out of 170), belonging to Clade 1. Most Clade 1 isolates were isolated at MSK. Half (8 out of 16) of the FM isolates are found in Clade 4. There are 49 highly similar isolates in Clade 1 and 19 highly similar isolates in Clade 4, all from patients at MSK. These may have originated recently from two single isolates, and they are indicated with blue triangles in Fig. 1. Some other isolates (e.g., MSK2233 and MSK2234 in Clade 2, both of which were isolated from the same patient [Table S1]) may also share a recent origin. However, overall, there is little evidence for geographical clustering. Each clade includes at least one isolate from both MSK and FM (Fig. 1). Three isolates from a clinical setting in Kuwait (40) are each located in a different clade (designated by Kw in Fig. 1). Interestingly, an environmental sample isolated from Irish soil, UCD321, groups with clinical isolates in Clade 5 from both MSK and FM (i.e., both the United States of America and Europe). The diversity of isolates obtained from the same clinical setting and the close relationship between isolates from different geographical settings highlight the global nature of C. parapsilosis as a human pathogen.

Only 9 of the 163 isolates that could be analyzed were aneuploid (one extra copy of chromosome 3, 4, 5, or 6) (Table S2A). Large segmental amplifications were also relatively rare, excluding telomeric and subtelomeric regions, which contained multiple variations in copy number (41, 42). We identified 11 amplifications and 5 deletions of >10 kb in size, 13 of which were found in only one isolate (Table S2B). Three large amplifications (from 125 to 250 kb) in three different isolates have a complex stair-step structure, similar to those described by Todd and Selmecki (10) in the azole-resistant isolates of C. albicans (Fig. S1). We also identified ~167 CNVs that are <10 kb in size, 85 of which are found in only one isolate (Table S2B). We further characterized two amplified regions with particularly interesting patterns. These are the only two amplifications that have occurred multiple times in multiple isolates and include an open reading frame.

10.1128/mbio.01777-22.3 TABLE S2 (A) Identification of aneuploid strains, (B) merged CNVs, and (C) all CNVs identified in all isolates. Download Table S2, XLSX file, 0.2 MB. Copyright © 2022 Bergin et al. 2022 Bergin et al. https://creativecommons.org/licenses/by/4.0/ This content is distributed under the terms of the Creative Commons Attribution 4.0 International license.

10.1128/mbio.01777-22.5 FIG S1 Identification of “stair-step” amplifications in C. parapsilosis. The structures of the large CNVs identified using DELLY were manually examined by plotting coverage levels. Three “stair-step” amplifications were identified. In these, an amplified central core is surrounded by two regions with lower copy numbers. The lower copy number regions are flanked by inverted repeat pairs (shown with arrows), which range in size from 1 kb to 5.4 kb. Download FIG S1, PDF file, 0.5 MB. Copyright © 2022 Bergin et al. 2022 Bergin et al. https://creativecommons.org/licenses/by/4.0/ This content is distributed under the terms of the Creative Commons Attribution 4.0 International license.

**FIG 2:**
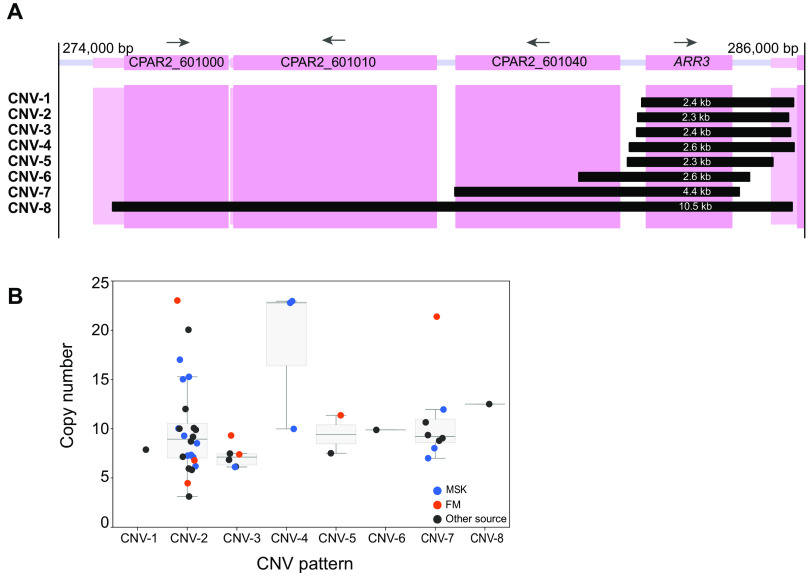
CNVs at the *ARR3* locus. (A) Span of the 8 different CNVs at the *ARR33* locus in C. parapsilosis. The coding sequences are shown by a dark pink box, and the flanking UTRs are shown in a lighter color. The extents of the tandemly repeating units in each of the 8 CNVs are shown by black boxes, labeled CNV-1 to CNV-8 on the left, and their lengths are indicated in white. Exact breakpoints were identified by interrogating split reads from the Illumina data, and the array structure of CNV-2 was verified by MinION sequencing of isolate UCD321. (B) Copy number of *ARR3* in each C. parapsilosis isolate. Each dot represents a single isolate: blue isolates from MSK, red from CHU de Nantes (FM), and dark gray from other sources. Medians and interquartile ranges are shown for CNVs present in more than one strain. Dots are jittered for clarity.

The first encompasses the gene *CPAR2_601050*, an ortholog of S. cerevisiae *ARR3*, an arsenite transporter (43). Amplifications of *ARR3* with different endpoints were previously described in four C. parapsilosis isolates (32). Pryszcz et al. (32) suggested that *ARR3* amplification may be induced in environmental conditions. *ARR3* is amplified in two of the three truly environmental (i.e., non-human-associated) isolates in our analysis (CBS1954, which was isolated from an olive tree in Italy, and UCD321, which was isolated from soil in Ireland [Table S1]) but not in the third (yHMJ4, which was isolated from berries in the United States [44] [Table S1]). In addition, we found *ARR3* amplifications in 44 other isolates associated with humans (Fig. 1; Table S1). In total, 8 different CNV patterns were identified with unique endpoints that ranged in size from 2.3 to 10.5 kb and had copy numbers ranging from 3 to 23 (Fig. 2; Table S1). Three CNVs extend into the adjacent gene *CPAR2_601040*, and one (previously described by Pryszcz et al. [32]) covers four genes (Fig. 2). An analysis of the strain phylogeny suggests that each amplification type arose only once (Fig. 1). The amplification of a telomeric gene cluster containing *ARR3* together with the transcription factor *ARR1* and the arsenate reductase *ARR2* has been previously reported in natural isolates of S. cerevisiae and Saccharomyces paradoxus (45), and a large subtelomeric amplicon that includes *ARR3* has been described in isolates from one clade of Cryptococcus neoformans var. *grubii* (46). Copy number correlates with resistance to arsenate. *ARR* genes are not clustered in *Candida* species, and *ARR3* is not located at the telomere.

**FIG 3:**
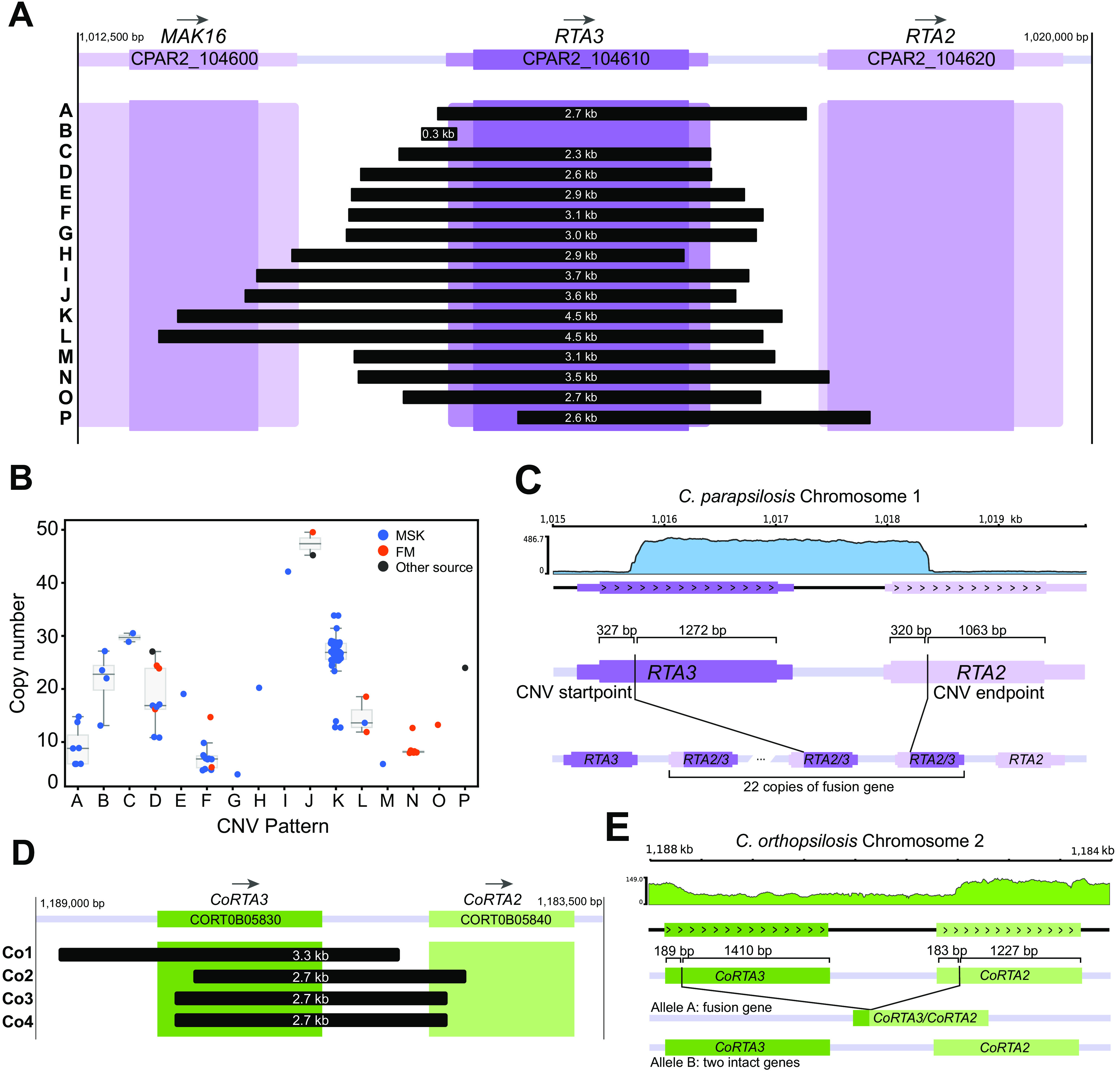
CNVs amplify different sequences at the *RTA3* locus. (A) Span of 16 different CNVs (from A to P) at the *RTA3* locus in C. parapsilosis. The coding sequence of *RTA3* is shown by a dark purple box, and the flanking UTRs are shown in a lighter color. The neighboring genes *MAK16* and *RTA2* are also shown. The extents of the tandemly repeating units in each of 16 CNVs are shown by black boxes, labeled (from A to P) on the left, and their lengths are indicated in white. Exact breakpoints were identified by interrogating split reads from the Illumina data, and the array structures of CNVs D and K were verified by MinION sequencing of isolates UCD321, MSK478, and MSK812. (B) Copy number of *RTA3* in each C. parapsilosis isolate. Each dot represents a single isolate: blue isolates from MSK, red from CHU de Nantes (FM), and dark gray from other sources. Medians and interquartile ranges are shown for CNVs present in more than one strain. Dots are jittered for clarity. (C) *RTA2/3* fusion gene in strain Kw3259-15 containing CNV-P. A coverage plot created by PyGenomeTracks (78) is shown at the top. The schematic shows the position of CNV breakpoints in relation to the CDS of both genes and the structure of the inferred array of fusion genes. (D) Amplifications of *RTA3* in C. orthopsilosis in strain 434. The orientation of the chromosome has been flipped to highlight the similarities to *RTA3* CNVs in C. parapsilosis. (E) A deletion in C. orthopsilosis generates a *CoRTA3/CoRTA2* fusion. A coverage plot of the locus is shown at the top. The schematic shows CNV breakpoints in both *CoRTA3* and *CoRTA2* and how the resulting fusion gene is formed at one allele while leaving the other allele intact.

The second amplification is linked to *RTA3* (*CPAR2_104610*). We previously showed that *RTA3*, encoding a putative PC floppase, had undergone extensive copy number amplification in 23 closely related C. parapsilosis isolates (33). Amplification was also observed in a small number of isolates by West et al. (47). We now show that the *RTA3* copy number is highly variable. It is increased in 104 of 170 isolates with multiple amplification patterns (Fig. 1 and 3).

### 16 unique CNVs amplify *RTA3*.

We found that *RTA3* has been amplified in 16 different types of CNV patterns, each with unique endpoints (each assigned a letter from A to P) (Fig. 1 and 3). Nine different CNV patterns were observed in isolates from Clade 1, whereas CNV-L is found only in isolates in Clade 3, and there are no CNVs in the Clade 2 isolates (Fig. 1). Most (15 out of 16) of the CNV patterns have a single evolutionary origin, and some are present in only a single isolate (Fig. 1). However, CNV-A may have originated three times (once in Clade 1 and twice in Clade 5).

To determine if the *RTA3* amplifications occur in tandem (and do not form extrachromosomal circles, such as those seen with *CUP1* [48]), we used MinION technology to sequence the genomes of 5 isolates (MSK478, MSK802, MSK803, MSK812, and UCD321), representing three *RTA3* CNV patterns (B, D, and K). In each case, some reads extended across part of the repeat unit and into the flanking DNA on each side of the repeat. This shows that at least for these isolates (and likely for all isolates), the repeats are in tandem on Chromosome 1 and are not extrachromosomal copies.

Thirteen CNV patterns result from tandem duplications that amplify the entire *RTA3* coding sequence (Fig. 3A), and one amplifies the promoter region only (CNV-B). The repeat units that include the *RTA3* ORF range in size from 2.3 to 4.5 kb. Four of these CNVs (CNV-I, J, K, and L) extend into the coding sequence of the upstream neighboring gene *MAK16*, and two (CNV-N and CNV-P) extend into the downstream gene *RTA2*. The copy number of *RTA3* varies both in isolates that share the same CNV pattern and in isolates with different CNV patterns (Fig. 3B). Isolate MSK807 (CNV-G) has the lowest estimated copy number of *RTA3* among the isolates with CNVs at four copies, whereas isolate FM16 (CNV-J) has the highest copy number at 50 copies.

For CNV-K, three isolates have roughly half the *RTA3* copy number of the other isolates with the same CNV pattern (~13× instead of ~26×) (Fig. 3B; Table S1). We considered the possibilities that only one allele of *RTA3* was amplified in these isolates (C. parapsilosis is diploid) and that both alleles were amplified in other isolates. We explored this issue by using long read sequencing (Oxford Nanopore) of C. parapsilosis MSK812, which is one of the CNV-K isolates with fewer copies of *RTA3* (estimated 14 copies) (Table S1). We found that it has 8 copies of *RTA3* at one allele and 6 copies at the other (Fig. S2). Similarly, the sequencing of isolate MSK478 (which also has CNV-K with ~25 copies of *RTA3*) showed that it has at least 11 copies at both alleles (Fig. S2B). Therefore, the variation in copy number among isolates with CNV-K is due to the expansion or contraction of the array in both alleles and is not due to hemizygosity for the amplification.

10.1128/mbio.01777-22.6 FIG S2 Copy number determination of *RTA3* CNV-K repeat. Visualizations of BLASTN results using the CNV-K repeat unit plus a 1 kb flanking sequence as a query against MinION reads for isolates MSK478 and MSK812, in which each plot represents the hits against a single read. Each line represents a hit, and adjacent hits are separated vertically for clarity. Read identifiers are shown on the *y* axis. (A) The exact copy number of CNV-K at both alleles was identified in isolate MSK812. (i) Seven reads in the MSK812 MinION dataset have 8 copies of the CNV-K repeat unit, and one is shown as an example. (ii) Eighteen reads in the MSK812 dataset have 6 copies of the CNV-K repeat unit, and one is shown as an example. (B) MSK478 has at least 11 copies on both alleles. No reads in the MSK478 dataset covered the entirety of the repeat array (i.e., no reads had a sequence matching both sides of the query flanking DNA). The read with the highest number of copies of the CNV-K repeat contained 11 copies, establishing a likely lower bound for the copy number at both alleles. Download FIG S2, PDF file, 0.3 MB. Copyright © 2022 Bergin et al. 2022 Bergin et al. https://creativecommons.org/licenses/by/4.0/ This content is distributed under the terms of the Creative Commons Attribution 4.0 International license.

Two of the *RTA3* CNVs alter the structure of the encoded protein. The amplification in CNV-P starts within the *RTA3* open reading frame and ends within the related adjacent gene *RTA2* (Fig. 3A and C). This repeat structure generates an array of in-frame *RTA2/RTA3* gene fusions, with the N terminus derived from *RTA2* and the C terminus derived from *RTA3* (Fig. 3C). *RTA3* and *RTA2* probably arose from an ancient duplication event, and both are present in many *Candida* clade species, including C. albicans (Fig. S3A). However, both *RTA2* and *RTA3* from the C. parapsilosis species complex are more closely related to C. albicans *RTA3* than either is to C. albicans *RTA2* (Fig. S3A). This likely resulted from a gene conversion event in the C. parapsilosis lineage. The sequences subsequently diverged, including an extension of the C terminus in Rta3 (532 aa) in C. parapsilosis that is not present in Rta2 (460 aa) (Fig. S3B). In CNV-H, the Rta3 protein is slightly truncated because this CNV consists of a tandem duplication with an endpoint 31 bp upstream of the stop codon of *RTA3*, resulting in a protein that is 10 amino acids shorter than its wild type counterpart.

10.1128/mbio.01777-22.7 FIG S3 Comparison of Rta2 and Rta3 proteins. (A) A phylogenetic tree was generated from MUSCLE alignments of Rta2 and Rta3 sequences from CUG-Ser species (CGOB) using PhyML, implemented in SeaView (80). The bootstrap values are shown. The pink box highlights the Rta2 and Rta3 sequences from the Candida parapsilosis complex. The gene names are taken from the Candida Gene Order Browser (http://cgob.ucd.ie). (B) Alignment of C. parapsilosis Rta2 and Rta3 generated using MUSCLE implemented in SeaView. Download FIG S3, PDF file, 0.5 MB. Copyright © 2022 Bergin et al. 2022 Bergin et al. https://creativecommons.org/licenses/by/4.0/ This content is distributed under the terms of the Creative Commons Attribution 4.0 International license.

CNV-B is substantially different from the others because it is an amplification of a small region (269 bp) that resides upstream and completely outside the *RTA3* coding sequence (Fig. 3A). Sequencing read coverage analysis suggested that the repeat has a complex organization in which direct repeats are interspersed with inverted copies of a central segment flanked by inverted-repeats, resulting in an *N*:(2*N*-1):*N* copy number pattern (Fig. S4A). This repeat structure was confirmed in MinION reads from isolates MSK802 and MSK803. The structure of the CNV-B repeat is similar to the DUP-TRP/INV-DUP structure seen in some human CNVs (30), although CNV-B repeats are much smaller and are reminiscent of amplifications formed via origin-dependent inverted repeat amplification (ODIRA) (9, 31). ODIRA results in complex CNVs with repeat units in head-to-head and tail-to-tail arrangements that are similar to the CNV-B structure. If CNV-B were caused by ODIRA, we would anticipate that the central region of the repeat would contain an origin of replication (Fig. S4B). However, we determined the temporal order of replication in C. parapsilosis using SORT-seq (49) (Fig. S4C), and we found no evidence that there is an origin near *RTA3*.

10.1128/mbio.01777-22.8 FIG S4 Structure of *RTA3* CNV-B. (A) (i) CNV-B consists of a central region (B) flanked by two regions (A and C) bounded by inverted repeat pairs (inward-facing triangles). The CNV occurs upstream of the *RTA3* coding sequence. (ii) CNV-B resolves as a repeat array of regions ABC interspersed with inverted copies of region B. (B) The ODIRA model of complex CNV generation, adapted from Brewer et al. (2015) (PLoS Genet 11:e1005699) under the terms of the Creative Commons Attribution License. The topmost diagram has been labeled to demonstrate the relationship to the observed CNV in (A). (C) Replication profile of strain MSK802 mapped to the C. parapsilosis reference genome. The relative DNA copy number, as a proxy for replication time, is on the *y* axis, where higher values denote earlier replication. The region containing *RTA3* on chromosome 1 is denoted by a red bar. Download FIG S4, PDF file, 1.4 MB. Copyright © 2022 Bergin et al. 2022 Bergin et al. https://creativecommons.org/licenses/by/4.0/ This content is distributed under the terms of the Creative Commons Attribution 4.0 International license.

We also looked for evidence of *RTA3* and *ARR3* amplification in other *Candida* species*. RTA3* is not amplified in 200 sequenced C. albicans genomes (50–53). However, we identified 4 different *RTA3* CNVs among 36 strains of Candida orthopsilosis (33, 52, 53) (Table S1), a close relative of C. parapsilosis (Fig. 3D). Only one of these (CNV-Co1) consists of a simple tandem amplification of the entire *CoRTA3* ORF. Two others, CNV-Co2 and CNV-Co3, amplify a fusion of the *CoRTA2* and *CoRTA3* genes, similar to CNV-P in C. parapsilosis (Fig. 3D). Interestingly, the last C. orthopsilosis CNV (CNV-Co4) involves a deletion. In the two strains containing CNV-Co4, the 3′ end of *CoRTA3*, the 5′ end of *CoRTA2*, and the intergenic space between them are deleted at one allele (Fig. 3E). This results in a new fusion gene with the N terminus derived from *CoRTA3* and the C terminus derived from *CoRTA2*. Single copies of *CoRTA2* and *CoRTA3* are intact on the other allele (Fig. 3E). This is the only example we have seen of an *RTA3* deletion in C. parapsilosis, C. orthopsilosis, or C. albicans. We did not observe the amplification of *ARR3* in C. albicans or C. orthopsilosis.

### The copy number of *RTA3* is correlated with miltefosine, but not fluconazole, resistance.

**FIG 4:**
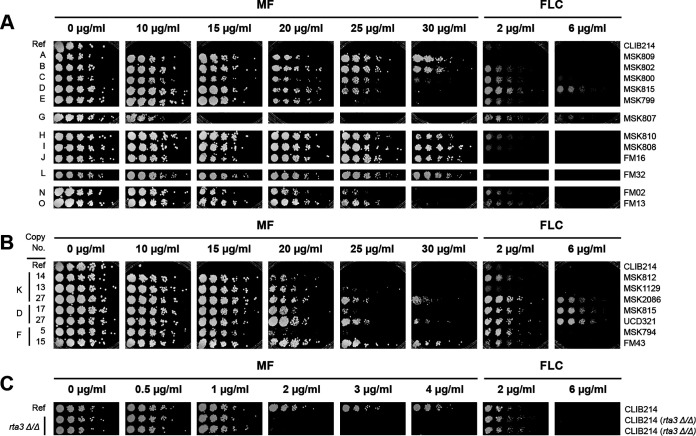
CNVs of *RTA3* correlate with resistance to miltefosine. (A) Multiple *RTA3* CNVs are associated with miltefosine resistance. A representative isolate of most CNV patterns (Fig. 2) was grown on YPD with increasing concentrations of miltefosine (MF) or fluconazole (FLC) as shown. Cells were serially diluted (1/5). The CNV pattern is indicated on the left, and the strain name is shown on the right. MF and FLC plates were incubated for 48 h. “Ref” indicates the reference strain, C. parapsilosis CLIB214. (B) Copy number is directly correlated with miltefosine resistance. Isolates with different copy numbers of CNV-K, CNV-D and CNV-F were grown as in (A). The *RTA3* copy number of each isolate is shown on the left. (C) Deleting *RTA3* reduces resistance to miltefosine. *RTA3* was deleted in C. parapsilosis CLIB214 using CRISPR-Cas9. The growth of two biological replicates is shown as in (A), except that the incubations on FLC were for 72 h.

The deletion of *RTA3* in C. albicans has been shown to increase susceptibility to miltefosine (38) and possibly to fluconazole (54). Therefore, we investigated the effect of copy number on the resistance of C. parapsilosis. Fig. 4A shows that the amplification of *RTA3* is associated with miltefosine resistance. For example, C. parapsilosis strains with only two copies of *RTA3* (one at each allele, e.g., the reference strain CLIB214) fail to grow at miltefosine concentrations of 10 μg/mL, whereas all of the strains with *RTA3* amplifications can grow at this concentration. Strains with CNVs A, H, I, J, and L can tolerate miltefosine concentrations up to at least 30 μg/mL, as can isolates with CNV-B (which amplifies only the region upstream of *RTA3*). The CNV with the weakest effect on MF resistance is CNV-G, which can tolerate 10 to 15 μg/mL, which is still higher than the tolerance of the reference strain CLIB214.

When different isolates carrying the same CNV pattern are compared, miltefosine resistance correlated with the copy number of *RTA3* (Fig. 4B). For example, C. parapsilosis isolates MSK812 and MSK1129, which have 13 to 14 copies of CNV-K, can tolerate a miltefosine concentration of up to ~20 μg/mL, whereas C. parapsilosis MSK2086, which has 27 copies, survives up to 30 μg/mL. Similarly, C. parapsilosis MSK815 with 17 copies of CNV-D and MSK794 with 5 copies of CNV-F tolerate miltefosine concentrations up to ~20 to 25 μg/mL, whereas UCD321 and FM43 with 27 and 15 copies of CNV-D and CNV-F, respectively, survive up to ~30 μg/mL (Fig. 4B).

Although the isolates have variable levels of sensitivity to fluconazole (Fig. 4), the copy number of *RTA3* does not correlate with susceptibility to this drug. For example, isolates MSK815 and UCD321, which have 17 and 27 copies of CNV-D, differ in their susceptibility to miltefosine, but they both tolerate fluconazole levels of 6 μg/mL.

We found that deleting *RTA3* in the reference isolate of C. parapsilosis (CLIB214) by CRISPR-Cas9 editing (55) results in an increased sensitivity to miltefosine (Fig. 4C). Low levels of miltefosine (up to 4 μg/mL) were used because the parental strain without any *RTA3* amplifications is highly sensitive. However, susceptibility to fluconazole was not affected by the deletion of *RTA3* (Fig. 4C).

Increasing the *RTA3* copy number correlates with increased expression, as shown by West et al. (47). However, we wondered whether amplifying the upstream region (CNV-B) has the same effect as amplifying the entire open reading frame. To explore this further, we measured RTA3 expression by reverse transcription polymerase chain reaction (RT-PCR) in one isolate of C. parapsilosis, MSK808, with approximately 42 copies of *RTA3* (CNV-I), and two other isolates (MSK802 and MSK803) in which only the promoter region was amplified (CNV-B). We found that *RTA3* expression is approximately 22-fold higher in C. parapsilosis MSK808 and is 2.8 to 6.6-fold higher in C. parapsilosis MSK802 and MSK803 than in the reference strain CLIB214 (Table 1). There are 28 copies of the direct repeat in CNV-B in C. parapsilosis MSK802 and 24 in C. parapsilosis MSK803. Thus, *RTA3* promoter region amplification (CNV-B) can also lead to increased expression.

**TABLE 1:** Expression of *RTA3* in strains with different amplifications

Isolate	Relative expression	Range	*P* value
MSK802 (CNV-B)	6.57	2.12 to 20.35	7.37E−03
MSK803 (CNV-B)	2.79	1.24 to 6.25	3.39E−02
MSK808 (CNV-I)	22.50	6.52 to 77.62	6.92E−05
CLIB214 (Reference)	1.00	0.19 to 5.27	NA

### Generation of miltefosine-resistant strains by experimental evolution.

**FIG 5:**
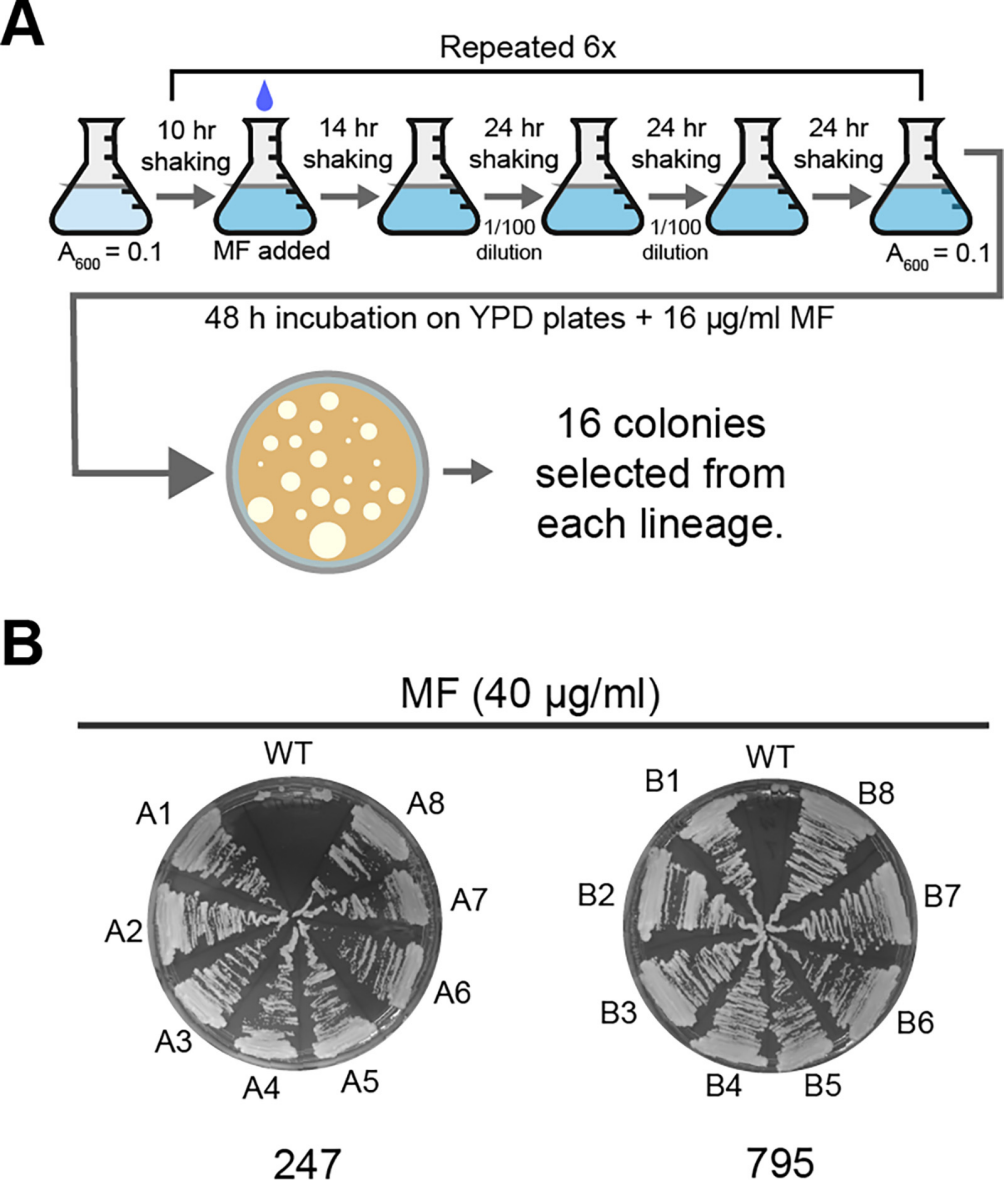
Generation of miltefosine-resistant C. parapsilosis isolates by adaptive laboratory evolution. (A) Six colonies (5 derived from C. parapsilosis MSK247 and one from C. parapsilosis MSK795) were inoculated in YPD at A_600_ = 0.1 and incubated at 30°C for 10 h. Miltefosine (indicated with a blue drop) was added to a final concentration of 1 μg/mL. The cultures were incubated for a further 14 h, then reinoculated into the same media using a 1/100 dilution, and incubated for 24 h. The dilution was repeated every 24 h for 3 days. The cells were then inoculated into fresh media and grown for 10 h, and then the miltefosine concentration was doubled. The process was repeated until the concentration of miltefosine reached 32 μg/mL (24 days). On day 25, the overnight cultures were plated on YPD with 16 μg/mL miltefosine. Sixteen colonies were picked from each lineage for further analysis, and the genomes of 10 of them were sequenced. (B) Growth of representative isolates evolved from C. parapsilosis MSK247 and C. parapsilosis MSK795 on 40 μg/mL miltefosine. WT = parental strain.

The prevalence of *RTA3* amplification in C. parapsilosis isolates suggests that there may be some strong selective pressure inducing amplification. To determine whether miltefosine was the driving force, we characterized the effect of exposing isolates to increasing concentrations of miltefosine in an adaptive laboratory evolution approach. We started with two isolates with only one copy of *RTA3* at each allele that were in the same clade as other isolates that had undergone amplification: C. parapsilosis MSK795 from Clade 1, which is related to isolates that have undergone 4 different CNVs (B, C, E, and F), and C. parapsilosis MSK247 from Clade 5, which is related to isolates with CNVs A, D, J, and M (Fig. 1). Five lineages were evolved from MSK247 (247A to 247E), and one was evolved from MSK795 (795B). Isolates were cultured in YPD with increasing concentrations of miltefosine, up to a maximum of 32 μg/mL, over a 26-day period (Fig. 5A). 16 randomly picked evolved colonies from each lineage tolerated miltefosine levels of 40 μg/mL (Fig. 4B). The genomes of 10 isolates derived from MSK247 and two from MSK795 were sequenced together with the parental strains. The sequenced strains are listed in the Materials and Methods section.

### Miltefosine-resistant isolates acquired homozygous disruptions in two flippase genes.

**FIG 6:**
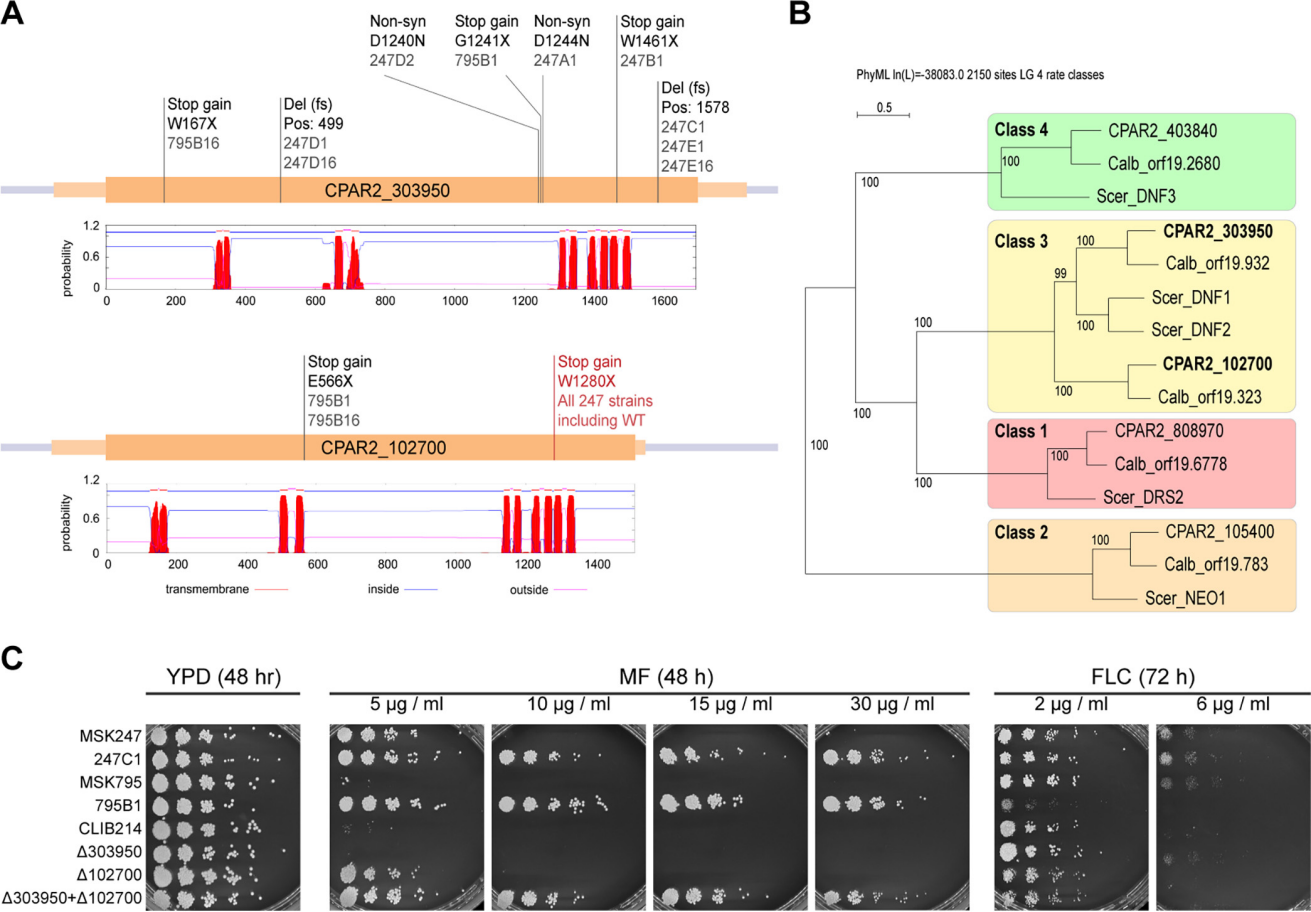
Protein-disrupting mutations in two flippase genes confer resistance to miltefosine in laboratory evolved strains. (A) Schematic showing homozygous mutations in P4-ATPases *CPAR2_102700* and *CPAR2_303950*. Mutations named in black were acquired during the laboratory evolution experiment in the evolved strains named below the mutation name. The mutation named in red is a natural homozygous variant found in the C. parapsilosis MSK247 isolate and in all strains derived from it. The predicted transmembrane topology of each protein (from TMHMM [79]) is shown, in which red peaks show predicted transmembrane domains. (B) Phylogeny of P4-ATPases in C. parapsilosis, C. albicans, and S. cerevisiae. The P4-ATPase genes in three yeast species were identified by BLAST and aligned, and a tree was constructed using SeaView (80). Monophyletic groups of genes were assigned to established classes (1–4, 81). Both *CPAR2_102700* and *CPAR2_303950* (in bold) belong to the Class 3 P4-ATPases. (C) Deleting *CPAR2_102700* and *CPAR2_303950* increases resistance to miltefosine but not to fluconazole. *CPAR2_102700* and *CPAR2_303950* were deleted singly or together in the C. parapsilosis CLIB214 background, and growth was compared to isolates evolved from C. parapsilosis MSK247 (247C1) and C. parapsilosis MSK795 (795B1) as in Fig. 5.

We did not find any evidence of the amplification of the *RTA3* locus in any of the evolved miltefosine-resistant strains. However, by comparing the sequences of the evolved strains to those of the parental strains, we identified two genes with homozygous loss-of-function variants in all 10 resistant isolates: *CPAR2_102700* and *CPAR2_303950*. These variants include frameshifts, nonsense mutations, and missense mutations that are predicted to be deleterious (Fig. 6A). *CPAR2_102700* and *CPAR2_303950* encode putative Class 3 P4-ATPases and are homologs of the PC flippase genes *DNF1* and *DNF2* in S. cerevisiae (Fig. 6B).

The two sequenced isolates derived from C. parapsilosis MSK795 (795B1 and 795B16) acquired mutations in both *CPAR2_102700* and *CPAR2_303950*, whereas the lineages derived from C. parapsilosis MSK247 acquired mutations only in *CPAR2_303950* (Fig. 6A). However, subsequent analysis revealed that C. parapsilosis MSK247 contains a homozygous natural variant in *CPAR2_102700*, resulting in a Trp-to-Stop nonsense mutation (W1280X) (Fig. 6A). C. parapsilosis MSK247 tolerates miltefosine concentrations of 5 μg/mL, whereas C. parapsilosis MSK795 fails to grow (Fig. 6C). Derivatives of both parents that carry homozygous inactivating mutations in both *CPAR2_303950* and *CPAR2_102700* can grow up to 30 μg/mL.

This result suggests that the reaching maximum level of miltefosine resistance requires the inactivation of both genes, *CPAR2_303950* and *CPAR2_102700*. To test this hypothesis, we deleted them, both separately and together, in the C. parapsilosis CLIB214 genetic background using CRISPR-Cas9 editing (Fig. 6C). Deleting *CPAR2_102700* alone in C. parapsilosis CLIB214 allows growth up to 5 μg/mL miltefosine, whereas deleting *CPAR2_303950* alone has no effect (Fig. 6C). However, strains in which both *CPAR2_303950* and *CPAR2_102700* were deleted tolerated at least 30 μg/mL miltefosine, similar to strains derived from the experimentally evolved isolates (MSK795B1, MSK247C1) (Fig. 6C). Deleting *CPAR2_102700* alone or in combination with *CPAR2_303950* slightly increases sensitivity to fluconazole (Fig. 6C).

## DISCUSSION

We identified two examples of CNVs in C. parapsilosis with unusual patterns that are likely to provide interesting models for studying amplification mechanisms. The amplifications occur in tandem, and, at least for *RTA3*, the copy number at each allele can vary. This suggests that the expansion and contraction of the CNV may occur by NAHR following misalignments at alleles with several gene copies. It is likely that the *RTA3/RTA2* gene fusion in CNV-P also originated by NAHR, due to the high sequence similarity between *RTA3* and *RTA2*. However, many C. parapsilosis isolates have only one copy of *RTA3* and *ARR3* at each allele (Fig. 1), meaning that some other mechanism must therefore underlie the initial amplification step. Possibilities include BIR and MMBIR, which are used to restart replication at collapsed replication forks. For example, a collision between RNA polymerase II at the *CUP1* promoter and replication from the adjacent origin may lead to fork collapse, which would be repaired by BIR (25, 56). The CNV-B pattern of *RTA3* amplification (in which only a short sequence upstream from the coding sequence is amplified) is particularly intriguing because it results in inversions and triplications of parts of the repeated sequence (Fig. S4A). This structure is reminiscent of the ODIRA model for CNV generation at *SUL1* in S. cerevisiae, in which replication errors at short inverted repeats result in the formation of an autonomously-replicating, extrachromosomal “dog bone” structure with inverted regions (31). These are proposed to subsequently reintegrate at *SUL1*, thereby forming a DUP-TRP/INV-DUP CNV in which some regions are duplicated (DUP) and others are triplicated (TRP), surrounding an inverted region. To date, ODIRA has only been observed in S. cerevisiae, and it is unclear whether it occurs in other yeasts (31).

Of the 14 CNVs resulting in the tandem array amplification of *RTA3*, only CNV-A and CNV-E have obvious repeat sequences at the CNV endpoints, and these are direct repeats of 19 bp and 10 bp, respectively. The other 12 have either no repeats or repeats of <5 bp (Fig. S5). The longer repeats at the breakpoints of CNV-A may explain why it has originated independently three times (Fig. 1). Short repeats can result in copy number variation from template switching in MMBIR (57). However, we did not find any evidence of a replication origin near *RTA3* or in the amplified sequence of the CNV-B pattern (Fig. S4C). This makes it unlikely that the amplification patterns A, B, or E use MMBIR or similar mechanisms to *CUP1* (fork stalling at repeats) or *SUL1* (ODIRA) in S. cerevisiae. No obvious mechanisms explain the CNVs without terminal repeats. However, even in *CUP1*, it is not clear whether the nearby origin of replication fires during a regular S phase (25), and it remains possible that a rarely used origin is present near *RTA3* or *ARR3* in C. parapsilosis.

10.1128/mbio.01777-22.9 FIG S5 Repeat sequences at the *RTA3* and *ARR3* amplifications. Diagram showing the sequences at the breakpoints for the (A) 16 *RTA3* CNVs and the (B) 8 *ARR3* CNVs. Breakpoints are demarcated by a switch from lowercase (outside the CNV) to uppercase (covered by the CNV) and vice-versa. Text highlighted in pink shows the similarity between the start points and the endpoints. Download FIG S5, PDF file, 0.4 MB. Copyright © 2022 Bergin et al. 2022 Bergin et al. https://creativecommons.org/licenses/by/4.0/ This content is distributed under the terms of the Creative Commons Attribution 4.0 International license.

We found that the copy number of *RTA3* correlates with resistance to miltefosine, a PC analog. This suggests that Rta3 controls the localization of PC. There is, however, some debate over whether Rta3-like proteins are transporters or signaling receptors. Rta3 is a member of the Rta1-family, which encodes proteins with 7 transmembrane domains, similar to the structure of G-protein-coupled receptors (GPCRs) (36). Within this family, Rta3 is more closely related to S. cerevisiae Rsb1 than to other members. Most of the early evidence suggested that Rsb1 directly flips LCBs in the plasma membrane (35). However, Johnson et al. (37) suggested that rather than acting as a transporter, Rsb1 determines resistance to the LCB phytosphingosine by regulating the endocytosis of the tryptophan transporter Tat2 either by signaling through a G-protein (as a GPCR) or by an arrestin-mediated effect on the targeting of ubiquitin ligases. In addition, Srivistava et al. (38) found that deleting *RTA3* in C. albicans resulted in the increased flipping of a fluorescently labeled PC, possibly by controlling the activity of an unknown flippase. An increase in copy number of *RTA3* and *RTA2* in C. albicans through chromosome duplication also affects tolerance to tunicamycin, an inducer of endoplasmic reticulum (ER) stress, though the exact mechanism is not known (58, 59).

Several pieces of evidence suggest that Rsb1 is a direct lipid transporter, specifically, a floppase. First, Rsb1 shares no sequence similarity with other yeast GPCRs (36). Second, Makuta et al. (60) showed that Rsb1 (and not other Rta1-family members) regulates LCB transport and that this activity is dependent on a loop region following TMS5 and not on the C terminus, as expected for a GPCR. Rsb1 in S. cerevisiae and Rta3 in C. albicans are located in the plasma membrane (34, 54). The increased flipping observed by Srivistava et al. (2017) (38) may be due to cross talk between sphingolipids and glycerophospholipids (e.g., PC), as shown in S. cerevisiae by Kihara and Igarashi (35). Changes in the distribution of one may be compensated by changing the distribution of the other (36). The association of *RTA3* copy number amplification with miltefosine resistance in C. parapsilosis is consistent with a role as a transporter. Our results also suggest that C. parapsilosis *CPAR2_102700* and *CPAR2_303950*, members of the class 3 family of P4-ATPases, are the direct flippases of PC. However, our data cannot rule out the alternative model of Johnson et al. (37), in which Rta3 regulates the functions of *CPAR2_102700* and *CPAR2_303950* rather than directly acting as a transporter.

Our results strongly suggest that there is selective pressure driving the amplification of *RTA3* and *ARR3* in C. parapsilosis. This conclusion is based on our observation that amplification occurred in many isolates that were from diverse genetic and environmental backgrounds and were distributed across the C. parapsilosis phylogeny (Fig. 1). We also found that there have been multiple independent events of amplification with at least 16 unique endpoints for *RTA3* and 8 for *ARR3*. The CNVs sometimes encompass parts of the adjacent genes, but *RTA3* and *ARR3* are the only complete genes amplified in all of them. There are some unusual features in *RTA3*. For example, in CNV-B, the likely promoter is amplified, and the 3′ is slightly truncated in the amplified copies in CNV-H. West et al. (47) previously described *RTA3* amplifications in 4 C. parapsilosis isolates, including one from the New York subway. Those amplifications also have different endpoints, but because the data come from metagenomics analyses, we cannot determine if their endpoints differ from those of the 16 CNVs we describe.

Most similar CNVs that have been described in other species occur during experimental adaptation to environmental conditions, including amplifications of *CUP1*, *SUL1*, *HXT6*, *GAP1*, and *DUR3* in S. cerevisiae (5, 7–9). From this group, only *CUP1* amplifications have been described in natural isolates. Other amplifications in natural isolates (including *ARR3*) occur in subtelomeric regions (45, 46). The majority of the C. parapsilosis isolates in this study are associated with humans (either clinical or from healthy donors), although one with an *RTA3* amplification (C. parapsilosis UCD321) and two with *ARR3* amplifications (CBS1954 and UCD321) have environmental origins (soil and olive tree) (Table S1). Whereas the *ARR3* amplifications may be induced by the presence of arsenate (45, 46), it is unlikely that the *RTA3* amplifications were driven by exposure to miltefosine because it is not a commonly used antifungal drug. In addition, as we have shown, exposure to miltefosine is likely to result in loss-of-function mutations in flippase genes. At present, we do not know what kind of selective pressure led to the widespread amplifications of *RTA3* in C. parapsilosis.

Miltefosine has potential as an antifungal drug (61–63), and it has recently been designated an orphan drug for the treatment of invasive candidiasis (https://www.accessdata.fda.gov/scripts/opdlisting/oopd/detailedIndex.cfm?cfgridkey=843921). However, our analyses suggest that it would be a poor choice, at least for invasive Candida parapsilosis infections. Many isolates are naturally resistant because of amplifications of *RTA3*, and in others, resistance rapidly arises due to loss-of-function mutations in the flippase genes. The resistance of Leishmania donovanai to miltefosine is also associated with mutations in flippases (64). Although mutations in two flippase genes are required for resistance in C. parapsilosis, we note that many of the C. parapsilosis samples that we examined (56 out of 170) have predicted loss-of-function variants in *CPAR2_102700*, meaning that acquiring a mutation in *CPAR2_303950* would be sufficient to render them highly resistant to miltefosine.

## MATERIALS AND METHODS

### Strains and growth.

The isolates used are listed in Table S1. The isolates were maintained on YPD agar (1% Bacto Yeast Extract [212750, Sigma], 2% Bacto Peptone [211677, Sigma], 2% Bacto Agar [214010, Sigma], 2% D-[+]-Glucose [G8270, Sigma]), and liquid cultures were grown in 5 mL YPD broth without agar at 30°C and 200 rpm shaking overnight. For serial dilutions, 0.5 mL of the overnight cultures were harvested at 13,000 rpm at room temperature for 1 min, washed twice in 0.5 mL of phosphate-buffered saline (PBS) buffer (BR0014G, Thermo Fisher), resuspended in 0.5 mL PBS, and diluted to A_600_ = 0.0625 (approximately 6.25 × 10^5^ cells/mL) in PBS. Five-fold serial dilutions were made in PBS and transferred with a pinner to YPD agar plates containing miltefosine (M5571, Sigma-Aldrich) or fluconazole (F8929, Sigma-Aldrich) at the indicated concentrations. The pinned plates were incubated at 30°C for the indicated times and photographed using a Singer PhenoBooth.

### Gene deletions/disruptions.

The entire open reading frames of *CPAR2_104610* (*RTA3*) and the flippases *CPAR2_303950* and *CPAR2_102700* were deleted using CRISPR-Cas9 with the pCP-tRNA system, as described in Lombardi et al. (55). All of the primers used for the gRNA and repair template synthesis are listed in Table S3.

10.1128/mbio.01777-22.4 TABLE S3 List of primers used. Download Table S3, DOCX file, 0.01 MB. Copyright © 2022 Bergin et al. 2022 Bergin et al. https://creativecommons.org/licenses/by/4.0/ This content is distributed under the terms of the Creative Commons Attribution 4.0 International license.

### Microevolution of miltefosine resistant isolates.

The microevolution method was modified from Papp et al. (65) and Ene et al. (66). Three colonies from C. parapsilosis MSK247 and C. parapsilosis MSK795 were originally chosen. Subsequent analysis showed that 5 evolved lineages were derived from C. parapsilosis MSK247 (247A to E) and that one was derived from C. parapsilosis MSK795 (795B). The colonies were incubated for 10 h in 5 mL YPD at 200 rpm and 30°C. Miltefosine was added to a final concentration of 1.0 μg/mL, and the cultures were incubated for a further 14 h. The cultures were diluted (1:100) into fresh media containing the same concentration of miltefosine every 24 h for 3 days. Overnight cultures were then diluted to A_600_ = 0.1 and incubated for 10 h, and the miltefosine concentration was doubled. This 5-day cycle was repeated until a concentration of 32 μg/mL of miltefosine was reached (Fig. 4A). The cultures were diluted and plated on YPD agar plates containing 16 μg/mL miltefosine, yielding 100 to 200 colonies on each plate, and were incubated at 30°C for 48 h. The genomes of 10 resistant isolates (247A1, 247B1, 247C1, 247D1, 247D2, 247D16,247E1, 247E16, 797B1, and 795B16), together with the parental strains, were sequenced by the Beijing Genomic Institute via DNBseq.

### Illumina sequencing.

Illumina sequencing of the MSK isolates was carried out as described in Zhai et al. (33). 45 C. parapsilosis isolates from Centre Hospitalier Universitaire de Nantes, France were screened for resistance to miltefosine, and 16 isolates were chosen for sequencing. Genomic DNA was isolated from 5 mL overnight cultures in YPD at 30°C using phenol-chloroform-isoamyl alcohol extraction. Cell pellets were resuspended in 200 μL of extraction buffer (Triton X-100 2% m/v, NaCl 100 mM, Tris 10 mM [pH 7.4], EDTA 1 mM, SDS 1% m/v) and transferred to screw cap tubes, and then approximately 0.3 g of acid-washed beads and 200 μL of phenol-chloroform-isoamyl alcohol (25:24:1) was added. Cells were lysed using a 1600 MiniG bead beater from Spex SamplePrep for 6 × 30 s with 30 s pauses in between and then centrifuged at 14,000 rpm for 10 min at room temperature. The supernatant was extracted twice more with 200 μL of TE, 200 μL of phenol-chloroform-isoamyl alcohol, and one 30 s agitation in the bead beater. DNA was precipitated using 80 μL of ammonium acetate (7.5 M) and 1 mL of 100% isopropanol, washed with 1 mL of 70% ethanol, and air-dried. The pellets were resuspended in 400 μL of TE with 1 μL of RNase A (100 mg/mL) and incubated overnight at 37°C. The DNA was precipitated again and resuspended in 150 μL water. Illumina sequencing was performed by the UCD Conway Genomics Core using a NextSeq 500. 1 ng of gDNA was tagmented (fragmented and tagged with adapter sequences) using the Nextera kit transposome. Dual-indexed paired-end libraries were prepared using the Nextera XT DNA Library Prep Kit. An Illumina NextSeq500 mid output 300 cycle sequencing kit was used to prepare and run the flowcell (HVGWJAFX2).

### Oxford Nanopore sequencing.

Strains were grown overnight in 50 mL of YPD broth, and genomic DNA was extracted from approximately 4 × 10^9^ cells using a Qiagen Genomic-tip 100/G kit (10223, Qiagen) with minor modifications. The lyticase incubation was extended to 2 h, and the proteinase K incubation was extended to overnight (~15 h). DNA libraries were prepared using three different kits as per the manufacturers’ instructions. Libraries from C. parapsilosis MSK812 and UCD321 were prepared using a Ligation Sequencing Kit (SQK-LSK109, Oxford Nanopore), using 1 μg of DNA per strain. DNA was repaired using a NEBNext FFPE DNA Repair Mix (M6630, New England Biolabs) and NEBNext Ultra II End repair/dA-tailing Module (E7546, New England Biolabs). Adapters were ligated using a NEBNext Quick Ligation Module (E6056, New England Biolabs). Libraries were sequenced on a MinION Mk1C device. Priming, loading and washing were performed using the EXP-FLP002, SQK-LSK109, and EXP-WSH002 Oxford Nanopore kits, respectively, as per the manufacturers’ instructions. The genomes were sequenced in parallel on one flow cell. The first isolate (C. parapsilosis MSK812) was sequenced for 24 h, and the flow cell was washed, reprimed, and loaded with the C. parapsilosis UCD321 isolate for an additional ~48 h. Libraries from C. parapsilosis MSK802 and MSK803 were prepared using a Rapid Barcode Sequencing Kit (SQK-RBK004, Oxford Nanopore), using 400 ng of DNA per sample. Samples were multiplexed using barcodes R04 and R05, respectively. Libraries were sequenced on an original MinION device for 72 h. Priming and loading were performed using the EXP-FLP002 and SQK-RBK004 kits, respectively. The C. parapsilosis MSK478 library was prepared using a Rapid Sequencing Kit (SQK-RAD004, Oxford Nanopore), using 400 ng of DNA, and sequenced for 72 h using an original MinION device. Basecalling was performed for all samples using guppy_basecaller with the following parameters: “–input_path fast5 –save_path fastq –c dna_r9.4.1_450bps_fast.cfg –verbose_logs –cpu_threads_per_caller 5 –num_callers 10”. Guppy v.4.2.2+effbaf8 was used for the MSK812 and UCD321 samples, and Guppy v.3.6 was used for the MSK802 and MSK803 samples. For the multiplexed samples MSK802 and MSK803, demultiplexing was performed using guppy_barcoder with the following parameters: “–barcode_kits SQK-RBK004 –t 30 –verbose_logs –trim_barcodes”. The reads were filtered using NanoFilt with the following parameters: “-l 1000 -q 7” (67).

### Sequence analysis.

The Illumina reads were trimmed with Skewer version 0.2.2 using tags “-m pe -t 4 -l 35 -q 30 -Q 30” (68). The trimmed reads were aligned to the C. parapsilosis reference genome using bwa-mem version 0.7.12. The resulting BAM files were sorted, and duplicate reads were marked using the GenomeAnalysisToolkit (GATK version 4.0.1.2) SortSam and MarkDuplicates tools, respectively. Variants were called using GATK HaplotypeCaller with the tag “–genotyping_mode DISCOVERY,” combined using GATK CombineGVCFs, and joint-genotyped using GATK GenotypeGVCFs. Variant files were filtered for read depth (<15) and genotype quality (<40) using GATK VariantFiltration. Additionally, clusters of SNPs (5 SNPs in a 100 bp window) were filtered using GATK VariantFiltration. A custom script was used to remove variants that were flanked on either side by a long string of mononucleotide or dinucleotide repeats and by variants that were called as heterozygous but had an allele depth ratio <0.25 or >0.75 (https://github.com/CMOTsean/milt_variant_filtration). Additionally, for tree construction, indels were excluded using GATK SelectVariants with the tag “–select-type-to-include SNP”. For the analysis of the evolved strains, a custom script was used to filter out variants in the evolved strains that were also present in the respective parent strain (https://github.com/CMOTsean/milt_variant_filtration).

### SIFT4G analysis.

A SIFT prediction database was created for C. parapsilosis using the SIFT4G algorithm and the recommended Uniref90 database as a reference for the protein sequences (69). The C. parapsilosis prediction database was used to annotate variants from the evolved strains with whether they are likely to be deleterious to protein function.

For each annotated gene in C. parapsilosis, the number of evolved strains which carried a variant predicted by SIFT to be protein function-affecting in that gene was tallied. Variants were also visualized using Integrative Genomics Viewer (IGV) to manually check results (70).

### Phylogeny construction.

Called SNPS were concatenated, and heterozygous sites were resolved randomly to either allele by 1,000 iterations of random repeated haplotype sampling (71). SNP trees were then constructed from each of the 1,000 haploid inputs using RAxML (v8.2.12) with the GTRGAMMA model of nucleotide substitution and the random number seed “-p 12345” (72). The tree with the highest maximum likelihood score was chosen, and the remaining 999 trees were used to generate branch support values.

### Estimating copy numbers.

Six strains were removed from the aneuploidy step because of uneven sequence coverage (full list used is in Table S2). The mean coverage of each chromosome (except the rDNA on Chromosome 7) for each of the remaining 163 strains was calculated using BEDTools and was divided by the average coverage of the genome to identify chromosome copy numbers. Chromosomes with copy numbers >2.5 were called as aneuploid.

CNVs were identified using DELLY (73) with default CNV length = 1,000 bp. CNVs within 20 kb of the telomeric regions, the rDNA, and the mitochondrial genome were removed (Table S2). Deletions which were over 1.25× coverage and duplications which were under 2.75× coverage were also removed. CNVs were merged if the start points or endpoints were within 1,000 bp. The lengths and coverages were averaged between all strains with the merged CNV (Table S2). Several strains with coverage problems were excluded from the merging step (FM05, FM06, FM07, FM10, FM14, FM32, FM43, 611, GA1_ERR246510, J931058, J931845, J950218, Kw1590-18, Kw2006-15, 103) because of uneven sequence coverage. For each strain with a CNV at *RTA3* or *ARR3*, the average coverage across the ORF in each isolate was found using BEDTools coverage (v2.29.2) (74). This value was divided by the average genome coverage (found with BEDTools genomecov) and multiplied by two to adjust for ploidy in order to calculate an estimate for the copy number. For CNVs that did not cover the entire *RTA3* ORF, the average coverage of a representative section of the CNV was used instead.

MinION read sequences were used to identify the exact *RTA3* copy number for a set of strains. The respective CNV sequences plus 1 kb flanking sequences on either side, were searched against the set of MinION reads for strains MSK478, MSK802, MSK803, MSK812, and UCD321 using BLASTN (v2.10.0). The search outputs were parsed with RECON-EBB (https://github.com/CMOTsean/recon-ebb) to estimate and visualize the copy number from reads which included hits for multiple copies of the CNV sequence and both regions of the flanking sequence (i.e., the reads which covered the entirety of the repeat region). The same method was used to characterize the *ARR3* amplification in UCD321.

### Replication timing profiles.

Relative replication time was determined by SORT-seq as described previously (42). Briefly, replicating (S phase) and nonreplicating (G_2_ phase) cells were enriched from an asynchronously growing culture by FACS based on DNA content. In each sample, genomic DNA was extracted and subjected to Illumina sequencing to measure the relative DNA copy number. Replication timing profiles were generated by normalizing the replicating (S phase) sample read count to the nonreplicating (G_2_) sample read count in 1 kb windows.

### Quantitative RT-PCR.

Cell harvesting and RNA extraction methods were adapted from Cravener and Mitchell (75). Cells were inoculated from overnight cultures to an A_600_ of 0.2 in 25 mL of prewarmed YPD broth and were incubated at 30°C for 6 h at 200 rpm using an orbital shaker. Cells were then harvested via vacuum filtration using MicroPlus-21 Sterile 0.45 μm filters (10407713, Whatman) and stored at −80°C for at least 24 h prior to RNA extraction. Cells were lysed by mechanical disruption as recommended in the Qiagen RNeasy Minikit (74104, Qiagen) with some modifications (75). Cells were lysed in RLT lysis buffer (Qiagen) and phenol-chloroform-isoamyl alcohol (25:24:1) (P3803, Sigma) in a 1:1 ratio. Lysis was performed using a 1600 MiniG from Spex SamplePrep using 30 s lysis followed by 30 s of chilling on ice for a total of 6 min. The RNA was extracted using a Qiagen RNeasy Minikit followed by two rounds of DNase digestion: one on-column using the Qiagen RNase-Free DNase Set (79254, Qiagen) and one off-column using Invitrogen’s TURBO DNA-free Kit (AM1907, Thermo Fisher). The cDNA was synthesized from 1 μg RNA using M-MLV reverse transcriptase (9PIM170, Promega) and Oligo(dT)15 primers (C110A, Promega), following the manufacturers’ instructions. Quantitative PCR was performed in 20 μL reactions using 50 ng cDNA using FastStart Universal SYBR green Master (Rox) (4913850001, Sigma) as per the manufacturers’ instructions on an Agilent Technologies Stratagene Mx3005p machine using default “two-step” settings. All primers are listed in Table S2. Relative quantification was performed using the 2(-Delta Delta C[T]) method by comparing the expression to *ACT1* and using C. parapsilosis CLIB214 as the calibrator strain. Calculations and statistics were performed in R using the pcr package (76).

### Data availability.

All sequencing data are deposited at NCBI under BioProject numbers PRJNA795920 and PRJNA748054 (SRP328964).
