# Supplementary material for: Systematic Analysis of Copy Number Variations in the Pathogenic Yeast Candida parapsilosis Identifies a Gene Amplification in RTA3 That is Associated with Drug Resistance
Source: mBio. 2022 Sep 19;13(5):e01777-22. doi: 10.1128/mbio.01777-22 (PMC9600344; doi:10.1128/mbio.01777-22)
Supplement: TABLE S1 [file mbio.01777-22-s0001.docx]

Table S1. List of strains

| **Strain** | | **Location** | **Origin**^1^ | **Species** | ***RTA3*** | | ***ARR3*** | | **Clade** | **Data Source** |
| --- | --- | --- | --- | --- | --- | --- | --- | --- | --- | --- |
|  | |  |  |  | **CNV type** | **Copy No.** | **CNV type** | **Copy No.** |  |  |
| 103 | | London, UK | Anus^2^ | *C. parapsilosis* | *-* | 2 | CNV-2 | 10 | 5 | This study |
| 02-203 | | Bergamo, Italy | Blood^2^ | *C. parapsilosis* | *-* | 2 | CNV-2 | 6 | 5 | This study |
| 73/037 | | Leeds, UK | Vagina^2^ | *C. parapsilosis* | *-* | 2 | - | 2 | 2 | This study |
| 73/107 | | London, UK | Mouth^2^ | *C. parapsilosis* | *-* | 2 | - | 2 | 2 | This study |
| 73/114 | | Leeds, UK | Anus^2^ | *C. parapsilosis* | *-* | 2 | CNV-2 | 10 | 5 | This study |
| 74/046 | | Leeds, UK | Aortic valve^2^ | *C. parapsilosis* | *-* | 2 | CNV-2 | 9 | 5 | This study |
| 81/040 | | London, UK | Toespace^2^ | *C. parapsilosis* | *-* | 2 | - | 2 | 5 | This study |
| 81/042 | | London, UK | Ear^2^ | *C. parapsilosis* | *-* | 2 | CNV-3 | 6 | 3 | This study |
| 81/253 | | London,UK | Nail^2^ | *C. parapsilosis* | *-* | 2 | CNV-2 | 3 | 5 | This study |
| 90-137 | | San Jose, USA | Orbital tissue^2^ | *C. parapsilosis* | *-* | 2 | CNV-2 | 6 | 5 | PRJNA563885 |
| BC014S | | Porto, Portugal | Blood | *C. parapsilosis* | - | 2 | - | 2 | 1 | PRJNA326748 |
| CBS1954 | | Italy | Environmental, olive tree | *C. parapsilosis* | - | 2 | CNV-6 | 10 | 2 | PRJEB1831 |
| CBS6318 | | USA | Healthy donor | *C. parapsilosis* | - | 2 | CNV-3 | 8 | 3 | PRJEB1831 |
| CDC173 | | Mississippi, USA | Blood or catheter | *C. parapsilosis* | - | 2 | CNV-7 | 9 | 4 | This study |
| CDC179 | | Mississippi, USA | Blood or catheter | *C. parapsilosis* | - | 2 | CNV-7 | 9 | 4 | This study |
| CDC317 | | Mississippi, USA | Hands (healthcare worker) | *C. parapsilosis* | - | 2 | CNV-7 | 9 | 4 | This study |
| CLIB214 | | Puerto Rico | Feces | *C. parapsilosis* | - | 2 | - | 2 | 2 | PRJNA563885 |
| CP35176 | | Unknown | Patient | *C. parapsilosis* | - | 2 | - | 2 | 2 | PRJNA361149 |
| CP35177 | | Unknown | Patient | *C. parapsilosis* | - | 2 | - | 2 | 2 | PRJNA361149 |
| GA1 | | Germany | Blood | *C. parapsilosis* | - | 2 | CNV-8 | 13 | 4 | PRJEB1831 |
| J931058 | | Belgium | Nail^2^ | *C. parapsilosis* | - | 2 | CNV-2 | 20 | 5 | This study |
| J931845 | | Japan | Unknown^2^ | *C. parapsilosis* | - | 2 | CNV-2 | 9 | 5 | This study |
| J950218 | | USA | Unknown^2^ | *C. parapsilosis* | - | 2 | CNV-3 | 7 | 3 | This study |
| J951066 | | Korea | Nail^2^ | *C. parapsilosis* | - | 2 | CNV-1 | 8 | 4 | This study |
| J961250 | | Lisbon, Portugal | Nail^2^ | *C. parapsilosis* | - | 2 | CNV-2 | 10 | 5 | This study |
| UCD321 | | Ireland | Environmental, soil | *C. parapsilosis* | D | 27 | CNV-2 | 12 | 5 | This study |
| yHMJ4 | | Plymouth MN, USA | Environmental, berries, leaves | *C. parapsilosis* | - | 2 | - | 2 | 1 | This study |
| YV1 | | Tuscany | Feces | *C. parapsilosis* | J | 45 | CNV-5 | 8 | 4 | PRJEB1831 |
| Kw1590-18 | | Kuwait | Patient | *C. parapsilosis* | - | 2 | CNV-7 | 11 | 4 | PRJNA661583 |
| Kw2006-15 | | Kuwait | Patient | *C. parapsilosis* | - | 2 | CNV-2 | 7 | 5 | PRJNA661583 |
| Kw3259-15 | | Kuwait | Patient | *C. parapsilosis* | Z | 24 | - | 2 | 1 | PRJNA661583 |
| FM02 | | Nantes, France | BAL fluid | *C. parapsilosis* | N | 8 | - | 2 | 4 | This study |
| FM03 | | Nantes, France | BAL fluid | *C. parapsilosis* | N | 8 | - | 2 | 4 | This study |
| FM05 | | Nantes, France | BAL fluid | *C. parapsilosis* | N | 8 | - | 2 | 4 | This study |
| FM06 | | Nantes, France | Peritoneal fluid | *C. parapsilosis* | F | 5 | - | 2 | 1 | This study |
| FM07 | | Nantes, France | Sputum | *C. parapsilosis* | D | 16 | CNV-2 | 23 | 5 | This study |
| FM10 | | Nantes, France | Anal swab | *C. parapsilosis* | N | 13 | - | 2 | 4 | This study |
| FM13 | | Nantes, France | Blood | *C. parapsilosis* | O | 13 | CNV-7 | 21 | 4 | This study |
| FM14 | | Nantes, France | Blood | *C. parapsilosis* | - | 2 | - | 2 | 2 | This study |
| FM16 | | Nantes, France | Bronchial secretions | *C. parapsilosis* | J | 50 | CNV-5 | 11 | 4 | This study |
| FM17 | | Nantes, France | BAL fluid | *C. parapsilosis* | N | 8 | - | 2 | 4 | This study |
| FM20 | | Nantes, France | Bronchial secretions | *C. parapsilosis* | N | 8 | - | 2 | 4 | This study |
| FM21 | | Nantes, France | Nail | *C. parapsilosis* | D | 24 | CNV-2 | 4 | 5 | This study |
| FM32 | | Nantes, France | Blood | *C. parapsilosis* | L | 19 | CNV-3 | 7 | 3 | This study |
| FM36 | | Nantes, France | Blood | *C. parapsilosis* | D | 24 | CNV-2 | 7 | 5 | This study |
| FM41 | | Nantes, France | Sputum | *C. parapsilosis* | L | 12 | CNV-2 | 9 | 3 | This study |
| FM43 | | Nantes, France | Finger nail secretions | *C. parapsilosis* | F | 15 | - | 2 | 1 | This study |
| MSK1 | | New York | Patient 12 | *C. parapsilosis* | K | 29 | - | 2 | 1 | PRJNA579121 |
| MSK1004 | | New York | Patient 13 | *C. parapsilosis* | K | 24 | - | 2 | 1 | This study |
| MSK1015 | | New York | Patient 13 | *C. parapsilosis* | K | 24 | - | 2 | 1 | This study |
| MSK1082 | | New York | Patient 11 | *C. parapsilosis* | D | 11 | CNV-2 | 15 | 5 | This study |
| MSK1090 | | New York | Patient 11 | *C. parapsilosis* | D | 11 | CNV-2 | 15 | 5 | This study |
| MSK1119 | | New York | Patient 1 | *C. parapsilosis* | K | 13 | - | 2 | 1 | This study |
| MSK1129 | | New York | Patient 1 | *C. parapsilosis* | K | 13 | - | 2 | 1 | This study |
| MSK1191 | | New York | Patient 2 | *C. parapsilosis* | - | 2 | - | 2 | 1 | This study |
| MSK1206 CP.20477.048 | | UM-Madison | Patient | *C. parapsilosis* | F | 7 | - | 2 | 1 | This study |
| MSK1241 | | New York | Patient 10 | *C. parapsilosis* | D | 17 | - | 2 | 5 | This study |
| MSK1258 | | New York | Patient 10 | *C. parapsilosis* | D | 17 | - | 2 | 5 | This study |
| MSK1286 | | New York | Patient 14 | *C. parapsilosis* | - | 2 | - | 2 | 4 | This study |
| MSK1298 | | New York | Patient 15 | *C. parapsilosis* | - | 2 | - | 2 | 4 | This study |
| MSK1302 | | New York | Patient 15 | *C. parapsilosis* | - | 2 | - | 2 | 4 | This study |
| MSK1324 | | New York | Patient 9 | *C. parapsilosis* | A | 6 | CNV-2 | 7 | 5 | This study |
| MSK1325 | | New York | Patient 9 | *C. parapsilosis* | A | 6 | CNV-2 | 7 | 5 | This study |
| MSK1351 | | Durham (Duke), NC | Patient | *C. parapsilosis* | - | 2 | - | 2 | 4 | This study |
| MSK1394 | | New York | Patient 5 | *C. parapsilosis* | F | 7 | - | 2 | 1 | This study |
| MSK1398 | | New York | Patient 5 | *C. parapsilosis* | F | 7 | - | 2 | 1 | This study |
| MSK1399 | | New York | Patient 5 | *C. parapsilosis* | F | 7 | - | 2 | 1 | This study |
| MSK158 | | New York | Patient 16 | *C. parapsilosis* | - | 2 | - | 2 | 4 | This study |
| MSK1617 | | Durham (Duke), NC | Patient | *C. parapsilosis* | - | 2 | - | 2 | 1 | This study |
| MSK1666 | | New York | Patient 13 | *C. parapsilosis* | - | 2 | - | 2 | 1 | This study |
| MSK17 | | New York | Patient 12 | *C. parapsilosis* | K | 29 | - | 2 | 1 | PRJNA579121 |
| MSK1700 | | New York | Patient 9 | *C. parapsilosis* | A | 6 | CNV-2 | 7 | 5 | This study |
| MSK1762 | | New York | Patient 17 | *C. parapsilosis* | K | 26 | - | 2 | 1 | This study |
| MSK18 | | New York | Patient 12 | *C. parapsilosis* | K | 27 | - | 2 | 1 | PRJNA579121 |
| MSK19 | | New York | Patient 12 | *C. parapsilosis* | K | 29 | - | 2 | 1 | PRJNA579121 |
| MSK2 | | New York | Patient 12 | *C. parapsilosis* | K | 29 | - | 2 | 1 | PRJNA579121 |
| MSK2049 | | New York | Patient 18 | *C. parapsilosis* | K | 27 | - | 2 | 1 | This study |
| MSK2057 | | New York | Patient 18 | *C. parapsilosis* | K | 27 | - | 2 | 1 | This study |
| MSK2060 | | New York | Patient 18 | *C. parapsilosis* | K | 28 | - | 2 | 1 | This study |
| MSK2084 | | New York | Patient 19 | *C. parapsilosis* | K | 27 | - | 2 | 1 | This study |
| MSK2086 | | New York | Patient 20 | *C. parapsilosis* | K | 27 | - | 2 | 1 | This study |
| MSK2092 | | New York | Patient 20 | *C. parapsilosis* | K | 27 | - | 2 | 1 | This study |
| MSK2094 | | New York | Patient 20 | *C. parapsilosis* | K | 27 | - | 2 | 1 | This study |
| MSK2107 | | New York | Patient 6 | *C. parapsilosis* | A | 9 | - | 2 | 1 | This study |
| MSK2108 | | New York | Patient 6 | *C. parapsilosis* | A | 9 | - | 2 | 1 | This study |
| MSK2123 | | New York | Patient 7 | *C. parapsilosis* | - | 2 | CNV-4 | 23 | 2 | This study |
| MSK2124 | | New York | Patient 7 | *C. parapsilosis* | - | 2 | CNV-4 | 23 | 2 | This study |
| MSK2131 | | New York | Patient 21 | *C. parapsilosis* | K | 28 | - | 2 | 1 | This study |
| MSK2134 | | New York | Patient 21 | *C. parapsilosis* | K | 29 | - | 2 | 1 | This study |
| MSK2141 | | New York | Patient 21 | *C. parapsilosis* | K | 27 | - | 2 | 1 | This study |
| MSK2159 | | New York | Patient 3 | *C. parapsilosis* | F | 5 | - | 2 | 1 | This study |
| MSK2160 | | New York | Patient 3 | *C. parapsilosis* | K | 27 | - | 2 | 1 | This study |
| MSK2161 | | New York | Patient 3 | *C. parapsilosis* | F | 5 | - | 2 | 1 | This study |
| MSK2162 | | New York | Patient 3 | *C. parapsilosis* | K | 27 | - | 2 | 1 | This study |
| MSK2191 | | New York | Patient 22 | *C. parapsilosis* | - | 2 | CNV-7 | 12 | 4 | This study |
| MSK2199 | | New York | Patient 22 | *C. parapsilosis* | A | 15 | CNV-2 | 6 | 5 | This study |
| MSK2233 | | New York | Patient 8 | *C. parapsilosis* | - | 2 | - | 2 | 2 | This study |
| MSK2234 | | New York | Patient 8 | *C. parapsilosis* | - | 2 | - | 2 | 2 | This study |
| MSK2248 | | New York | Patient 23 | *C. parapsilosis* | K | 27 | - | 2 | 1 | This study |
| MSK2384 | | New York | Patient 24 | *C. parapsilosis* | L | 14 | CNV-3 | 6 | 3 | This study |
| MSK2386 | | New York | Patient 25 | *C. parapsilosis* | - | 2 | - | 2 | 2 | This study |
| MSK2387 | | New York | Patient 26 | *C. parapsilosis* | B | 22^2^ | - | 2 | 1 | This study |
| MSK2389 | | New York | Patient 27 | *C. parapsilosis* | B | 13^2^ | - | 2 | 1 | This study |
| MSK2390 | | New York | Patient 28 | *C. parapsilosis* | - | 2 | - | 2 | 1 | This study |
| MSK247 | | New York | Patient 29 | *C. parapsilosis* | - | 2 | CNV-2 | 9 | 5 | PRJNA579121 |
| MSK249 | | New York | Patient 29 | *C. parapsilosis* | - | 2 | - | 2 | 4 | PRJNA579121 |
| MSK250 | | New York | Patient 29 | *C. parapsilosis* | - | 2 | - | 2 | 4 | PRJNA579121 |
| MSK251 | | New York | Patient 29 | *C. parapsilosis* | - | 2 | - | 2 | 4 | PRJNA579121 |
| MSK264 | | New York | Patient 29 | *C. parapsilosis* | K | 25 | - | 2 | 1 | PRJNA579121 |
| MSK265 | | New York | Patient 29 | *C. parapsilosis* | - | 2 | - | 2 | 4 | PRJNA579121 |
| MSK266 | | New York | Patient 29 | *C. parapsilosis* | - | 2 | - | 2 | 4 | PRJNA579121 |
| MSK281 | | New York | Patient 29 | *C. parapsilosis* | - | 2 | - | 2 | 4 | PRJNA579121 |
| MSK282 | | New York | Patient 29 | *C. parapsilosis* | K | 28 | - | 2 | 1 | PRJNA579121 |
| MSK283 | | New York | Patient 29 | *C. parapsilosis* | - | 2 | - | 2 | 4 | PRJNA579121 |
| MSK296 | | New York | Patient 29 | *C. parapsilosis* | K | 31 | - | 2 | 1 | PRJNA579121 |
| MSK297 | | New York | Patient 29 | *C. parapsilosis* | K | 29 | - | 2 | 1 | PRJNA579121 |
| MSK298 | | New York | Patient 29 | *C. parapsilosis* | K | 29 | - | 2 | 1 | PRJNA579121 |
| MSK313 | | New York | Patient 29 | *C. parapsilosis* | K | 27 | - | 2 | 1 | PRJNA579121 |
| MSK314 | | New York | Patient 29 | *C. parapsilosis* | K | 34 | - | 2 | 1 | PRJNA579121 |
| MSK315 | | New York | Patient 29 | *C. parapsilosis* | K | 34 | - | 2 | 1 | PRJNA579121 |
| MSK33 | | New York | Patient 12 | *C. parapsilosis* | K | 26 | - | 2 | 1 | PRJNA579121 |
| MSK34 | | New York | Patient 12 | *C. parapsilosis* | K | 26 | - | 2 | 1 | PRJNA579121 |
| MSK35 | | New York | Patient 12 | *C. parapsilosis* | K | 29 | - | 2 | 1 | PRJNA579121 |
| MSK478 | | New York | Patient 29 | *C. parapsilosis* | K | 25 | - | 2 | 1 | PRJNA579121 |
| MSK485 | | New York | Patient 2 | *C. parapsilosis* | K | 25 | - | 2 | 1 | This study |
| MSK486 | | New York | Patient 2 | *C. parapsilosis* | K | 29 | - | 2 | 1 | This study |
| MSK489 | | New York | Patient 4 | *C. parapsilosis* | F | 7 | - | 2 | 1 | This study |
| MSK49 | | New York | Patient 12 | *C. parapsilosis* | K | 29 | - | 2 | 1 | PRJNA579121 |
| MSK490 | | New York | Patient 4 | *C. parapsilosis* | F | 8 | - | 2 | 1 | This study |
| MSK5 | | New York | Patient 12 | *C. parapsilosis* | K | 29 | - | 2 | 1 | PRJNA579121 |
| MSK51 | | New York | Patient 12 | *C. parapsilosis* | K | 25 | - | 2 | 1 | PRJNA579121 |
| MSK519 | | New York | Patient 2 | *C. parapsilosis* | K | 25 | - | 2 | 1 | This study |
| MSK52 | | New York | Patient 12 | *C. parapsilosis* | K | 27 | - | 2 | 1 | PRJNA579121 |
| MSK520 | | New York | Patient 2 | *C. parapsilosis* | K | 26 | - | 2 | 1 | This study |
| MSK522 | | New York | Patient 2 | *C. parapsilosis* | K | 25 | - | 2 | 1 | This study |
| MSK536 | | New York | Patient 2 | *C. parapsilosis* | K | 27 | - | 2 | 1 | This study |
| MSK543 | | New York | Patient 2 | *C. parapsilosis* | K | 26 | - | 2 | 1 | This study |
| MSK544 | | New York | Patient 2 | *C. parapsilosis* | K | 23 | - | 2 | 1 | This study |
| MSK608 | | New York | Patient 30 | *C. parapsilosis* | - | 2 | - | 2 | 4 | This study |
| MSK611 | | New York | Patient 30 | *C. parapsilosis* | - | 2 | - | 2 | 4 | This study |
| MSK612 | | New York | Patient 30 | *C. parapsilosis* | - | 2 | - | 2 | 4 | This study |
| MSK620 | | New York | Patient 2 | *C. parapsilosis* | - | 2 | - | 2 | 1 | This study |
| MSK624 | | New York | Patient 2 | *C. parapsilosis* | K | 26 | - | 2 | 1 | This study |
| MSK630 | | New York | Patient 2 | *C. parapsilosis* | K | 26 | - | 2 | 1 | This study |
| MSK65 | | New York | Patient 12 | *C. parapsilosis* | K | 27 | - | 2 | 1 | PRJNA579121 |
| MSK67 | | New York | Patient 12 | *C. parapsilosis* | K | 29 | - | 2 | 1 | PRJNA579121 |
| MSK794 | | New York | Patient 3 | *C. parapsilosis* | F | 5 | - | 2 | 1 | This study |
| MSK795 | | New York | Patient 22 | *C. parapsilosis* | - | 2 | - | 2 | 1 | This study |
| MSK799 | | New York | Patient 31 | *C. parapsilosis* | E | 19 | - | 2 | 1 | This study |
| MSK800 | | New York | Patient 32 | *C. parapsilosis* | C | 29 | - | 2 | 1 | This study |
| MSK802 | | New York | Patient 33 | *C. parapsilosis* | B | 27^3^ | - | 2 | 1 | This study |
| MSK803 | | New York | Patient 34 | *C. parapsilosis* | B | 24^3^ | - | 2 | 1 | This study |
| MSK804 | | New York | Patient 30 | *C. parapsilosis* | - | 2 | - | 2 | 4 | This study |
| MSK806 | | New York | Patient 35 | *C. parapsilosis* | F | 10 | - | 2 | 1 | This study |
| MSK807 | | New York | Patient 36 | *C. parapsilosis* | G | 4 | CNV-2 | 2 | 5 | This study |
| MSK808 | | New York | Patient 37 | *C. parapsilosis* | I | 42 | - | 2 | 1 | This study |
| MSK809 | | New York | Patient 38 | *C. parapsilosis* | A | 14 | - | 2 | 5 | This study |
| MSK810 | | New York | Patient 39 | *C. parapsilosis* | H | 19 | - | 2 | 1 | This study |
| MSK811 | | New York | Patient 40 | *C. parapsilosis* | - | 2 | CNV-4 | 2 | 2 | This study |
| MSK812 | | New York | Patient 41 | *C. parapsilosis* | K | 14 | - | 2 | 1 | This study |
| MSK813 | | New York | Patient 42 | *C. parapsilosis* | C | 31 | - | 2 | 1 | This study |
| MSK814 | | New York | Patient 43 | *C. parapsilosis* | - | 2 | - | 2 | 3 | This study |
| MSK815 | | New York | Patient 44 | *C. parapsilosis* | D | 17 | CNV-2 | 2 | 5 | This study |
| MSK844 | | New York | Healthy donor 1 | *C. parapsilosis* | - | 2 | - | 2 | 2 | This study |
| MSK846 | | New York | Healthy donor 1 | *C. parapsilosis* | M | 6 | CNV-2 | 9 | 5 | This study |
| MSK848 | | New York | Healthy donor 2 | *C. parapsilosis* | - | 2 | CNV-7 | 8 | 4 | This study |
| MSK850 | | New York | Healthy donor 2 | *C. parapsilosis* | - | 2 | CNV-7 | 7 | 4 | This study |
| MSK891 | | New York | Patient 45 | *C. parapsilosis* | - | 2 | - | 2 | 4 | This study |
| MSK923 | | New York | Patient 45 | *C. parapsilosis* | - | 2 | - | 2 | 4 | This study |
| MSK925 | | New York | Patient 45 | *C. parapsilosis* | - | 2 | - | 2 | 4 | This study |
| 151 (CAS08-0151) | | Atlanta, USA | Blood | *C. orthopsilosis* | Co2 | 13 | - | 2 | N/A | PRJNA322245 |
| 1540 (CAS09-1540) | | Baltimore, USA | Blood | *C. orthopsilosis* | Co3 | 19 | - | 2 | N/A | PRJNA322245 |
| 1799 (CAS09-1799) | | Atlanta, USA | Blood | *C. orthopsilosis* | Co2 | 24 | - | 2 | N/A | PRJNA322245 |
| 1825 (CAS10-1825) | | Baltimore, USA | Blood | *C. orthopsilosis* | Co2 | 15 | - | 2 | N/A | PRJNA322245 |
| 185 (CAS08-0185) | | Atlanta, USA | Blood | *C. orthopsilosis* | Co2 | 13 | - | 2 | N/A | PRJNA322245 |
| 282 (CAS08-0282) | | Baltimore, USA | Blood | *C. orthopsilosis* | - | 2 | - | 2 | N/A | PRJNA322245 |
| 320 (CAS08-0320) | | Baltimore, USA | Blood | *C. orthopsilosis* | - | 2 | - | 2 | N/A | PRJNA322245 |
| 421 (CP25) | | Pisa, Italy | Nail^2^ | *C. orthopsilosis* | Co2 | 19 | - | 2 | N/A | PRJNA322245 |
| 422 (CP47) | | Pisa, Italy | Skin^2^ | *C. orthopsilosis* | Co3 | 21 | - | 2 | N/A | PRJNA322245 |
| 423 (CP85) | | L’Aquila, Italy | Catheter^2^ | *C. orthopsilosis* | Co2 | 39 | - | 2 | N/A | PRJNA322245 |
| 424 (CP124) | | Pisa, Italy | Bronchial aspirate^2^ | *C. orthopsilosis* | - | 2 | - | 2 | N/A | PRJNA322245 |
| 425 (CP125) | | Pisa, Italy | Nail^2^ | *C. orthopsilosis* | Co3 | 18 | - | 2 | N/A | PRJNA322245 |
| 426 (CP185) | | Varese, Italy | Blood^2^ | *C. orthopsilosis* | - | 2 | - | 2 | N/A | PRJNA322245 |
| 427 (CP269) | | Pisa, Italy | Bronchial aspirate^2^ | *C. orthopsilosis* | - | 2 | - | 2 | N/A | PRJNA322245 |
| 428 (CP287) | | Hong Kong | Toenail^2^ | *C. orthopsilosis* | - | 2 | - | 2 | N/A | PRJNA322245 |
| 433 (CP288) | | St. Niklaas, Belgium | Nail^2^ | *C. orthopsilosis* | - | 2 | - | 2 | N/A | PRJNA322245 |
| 434 (CP289) | | NCPF, UK | Unknown^2^ | *C. orthopsilosis* | Co4 | 1 | - | 2 | N/A | PRJNA322245 |
| 435 (CP296) | | Pisa, Italy | Skin^2^ | *C. orthopsilosis* | - | 2 | - | 2 | N/A | PRJNA322245 |
| 436 (CP331) | | Pisa, Italy | Sputum^2^ | *C. orthopsilosis* | - | 2 | - | 2 | N/A | PRJNA322245 |
| 437 (CP344) | | Pisa, Italy | Catheter^2^ | *C. orthopsilosis* | Co3 | 19 | - | 2 | N/A | PRJNA322245 |
| MSK477 | | New York | Patient 29 | *C. orthopsilosis* | Co1 | 8 | - | 2 | N/A | PRJNA579121 |
| MSK479 | | New York | Patient 29 | *C. orthopsilosis* | Co1 | 9 | - | 2 | N/A | PRJNA579121 |
| 498 (CAS08-0498) | | Baltimore, USA | Blood | *C. orthopsilosis* | - | 2 | - | 2 | N/A | PRJNA322245 |
| 504 (CAS08-0504) | | Baltimore, USA | Blood | *C. orthopsilosis* | Co1 | 9 | - | 2 | N/A | PRJNA322245 |
| 599 (CAS08-0599) | | Baltimore, USA | Blood | *C. orthopsilosis* | Co2 | 20 | - | 2 | N/A | PRJNA322245 |
| MSK616 | | New York | Patient 46 | *C. orthopsilosis* | - | 8^4^ | - | 2 | N/A | This study |
| MSK636 | | New York | Patient 29 | *C. orthopsilosis* | Co1 | 8 | - | 2 | N/A | PRJNA579121 |
| MSK638 | | New York | Patient 29 | *C. orthopsilosis* | Co1 | 8 | - | 2 | N/A | PRJNA579121 |
| MSK639 | | New York | Patient 29 | *C. orthopsilosis* | Co1 | 9 | - | 2 | N/A | PRJNA579121 |
| 748 (CAS09-0748) | | Atlanta, USA | Blood | *C. orthopsilosis* | - | 2 | - | 2 | N/A | PRJNA322245 |
| MSK805 | | New York | Patient 46 | *C. orthopsilosis* | - | 2 | - | 2 | N/A | This study |
| 831 (CAS09-0831) | | Baltimore, USA | Blood | *C. orthopsilosis* | Co1 | 9 | - | 2 | N/A | PRJNA322245 |
| 90-125 | | San Francisco, USA | Unknown | *C. orthopsilosis* | - | 2 | - | 2 | N/A | PRJNA431439 |
| B-8274 | | Pakistan | Unknown | *C. orthopsilosis* | - | 2 | - | 2 | N/A | PRJNA322245 |
| B-8323 | | Pakistan | Unknown | *C. orthopsilosis* | - | 2 | - | 2 | N/A | PRJNA322245 |
| MCO456 | | San Antonio, USA | Unknown | *C. orthopsilosis* | Co4 | 1 | - | 2 | N/A | PRJEB4430 |
|  | Engineered/evolved strains | | | | | | | | | |
| Name | | Parent |  | Description | | | | | | |
| 795B, B16 | | MSK795 |  | Miltefosine-resistant evolved strains | | | | | | |
| 247A1,B1,C1,D1,D2, D16,E1, E16 | | MSK247 |  | Miltefosine-resistant evolved strains | | | | | | |
| rta3*Δ*/*Δ* 1 | | CLIB214 |  | *rta3*Δ/*rta3*Δ (*cpar2_104610Δ* / *cpar2_104610*Δ) | | | | | | |
| rta3*Δ*/*Δ* 2 | | CLIB214 |  | *rta3*Δ/*rta3*Δ (*cpar2_104610Δ* / *cpar2_104610*Δ) | | | | | | |
| CPAR2_102700*Δ*/*Δ* 1 | | CLIB214 |  | *cpar2_102700Δ* / *cpar2_102700Δ* | | | | | | |
| CPAR2_303950*Δ*/*Δ* 1 | | CLIB214 |  | *cpar2_303950Δ* / *cpar2_303950Δ* | | | | | | |
| CPAR2_303950*Δ*/*Δ*, CPAR2_102700*Δ*/*Δ* | | CLIB214 |  | *cpar2_102700Δ* / *cpar2_102700Δ, cpar2_303950Δ* / *cpar2_303950Δ* | | | | | | |

^1^For MSK isolates, newly reported strains isolated from the same patient (where known) are indicated using random patient numbers. Four were isolated from fecal samples from two healthy donors at MSK. All FM strains were isolated from different patients. ^2^Information from Tavanti et al (2005) J. Clin Micro 43:284-292 and Tavanti et al (2007) J. Clin Micro 45:1455-1462. Known environmental isolates are indicated.

^3^Copy number for strains with CNV-B was measured as number of copies of full ABC repeat (Supp. Fig 2)

^4^Sequence coverage data for this strain was too poor quality to identify CNV
