# Supplementary material for: Systematic Analysis of Copy Number Variations in the Pathogenic Yeast Candida parapsilosis Identifies a Gene Amplification in RTA3 That is Associated with Drug Resistance
Source: mBio. 2022 Sep 19;13(5):e01777-22. doi: 10.1128/mbio.01777-22 (PMC9600344; doi:10.1128/mbio.01777-22)
Supplement: FIG S1 [file mbio.01777-22-s0004.pdf]

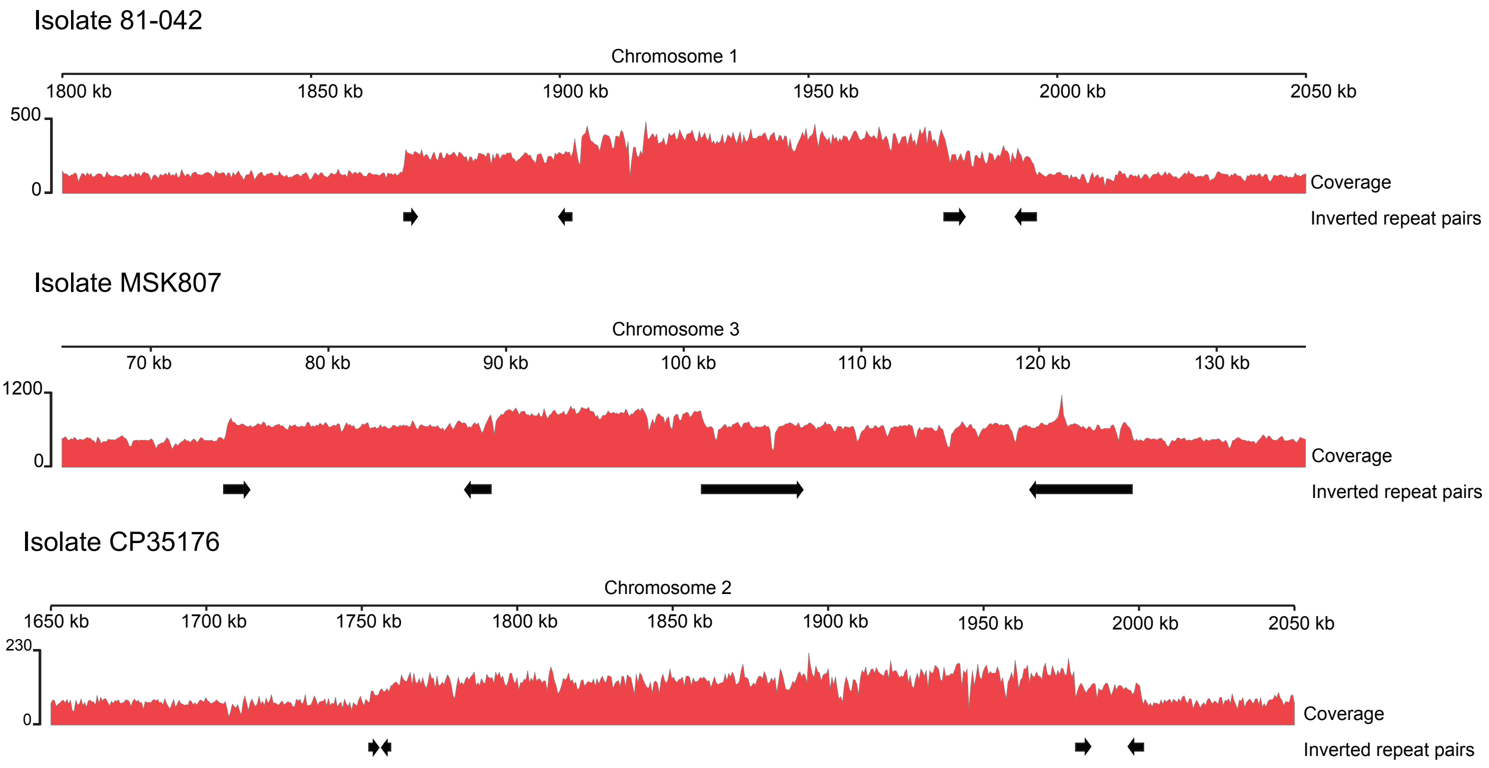

Supplementary Figure 1. Identification of “stair-step” amplifications in *C. parapsilosis*.

The structure of large CNVs identified using DELLY were manually examined by plotting coverage levels. Three “stair-step” amplifications were identified, in which an amplified central core is surrounded by two regions with lower copy number. The lower copy number regions are flanked by inverted repeats (shown with arrows), which range in size from 1 kb to 5.4 kb.
