# Supplementary material for: Systematic Analysis of Copy Number Variations in the Pathogenic Yeast Candida parapsilosis Identifies a Gene Amplification in RTA3 That is Associated with Drug Resistance
Source: mBio. 2022 Sep 19;13(5):e01777-22. doi: 10.1128/mbio.01777-22 (PMC9600344; doi:10.1128/mbio.01777-22)
Supplement: FIG S2 [file mbio.01777-22-s0005.pdf]

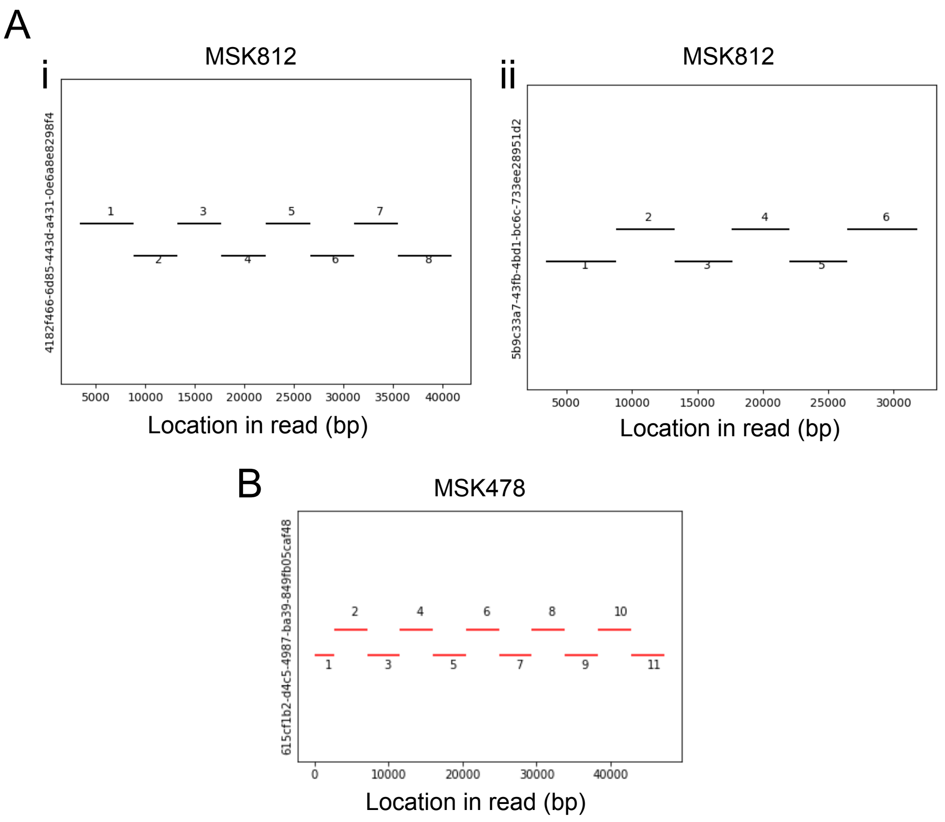

Supplementary Figure S2. Copy number determination of CNV-K repeat.

Visualizations of BLASTN results using the CNV-K repeat unit plus 1 kb flanking sequence as query against MinION reads for isolates MSK478 and MSK812 where each plot represents hits against a single read. Each line represents a hit, and adjacent hits are separated vertically for clarity. Read identifiers are shown on the y-axis.

A. The exact copy number of CNV-K at both alleles was identified in isolate MSK812. (i) Seven reads in the MSK812 MinION dataset have 8 copies of the CNV-K repeat unit, one is shown as an example. (ii) Eighteen reads in the MSK812 dataset have 6 copies of the CNV-K repeat unit, one is shown as an example.

B. MSK478 has at least 11 copies on both alleles. No reads in the MSK478 data-set covered the entirety of the repeat array, i.e. no reads had sequence matching both sides of the query flanking DNA. The read with the highest number of copies of the CNV-K repeat contains 11 copies, establishing a likely lower bound for copy number at both alleles.
