# Supplementary material for: Systematic Analysis of Copy Number Variations in the Pathogenic Yeast Candida parapsilosis Identifies a Gene Amplification in RTA3 That is Associated with Drug Resistance
Source: mBio. 2022 Sep 19;13(5):e01777-22. doi: 10.1128/mbio.01777-22 (PMC9600344; doi:10.1128/mbio.01777-22)
Supplement: FIG S3 [file mbio.01777-22-s0006.pdf]

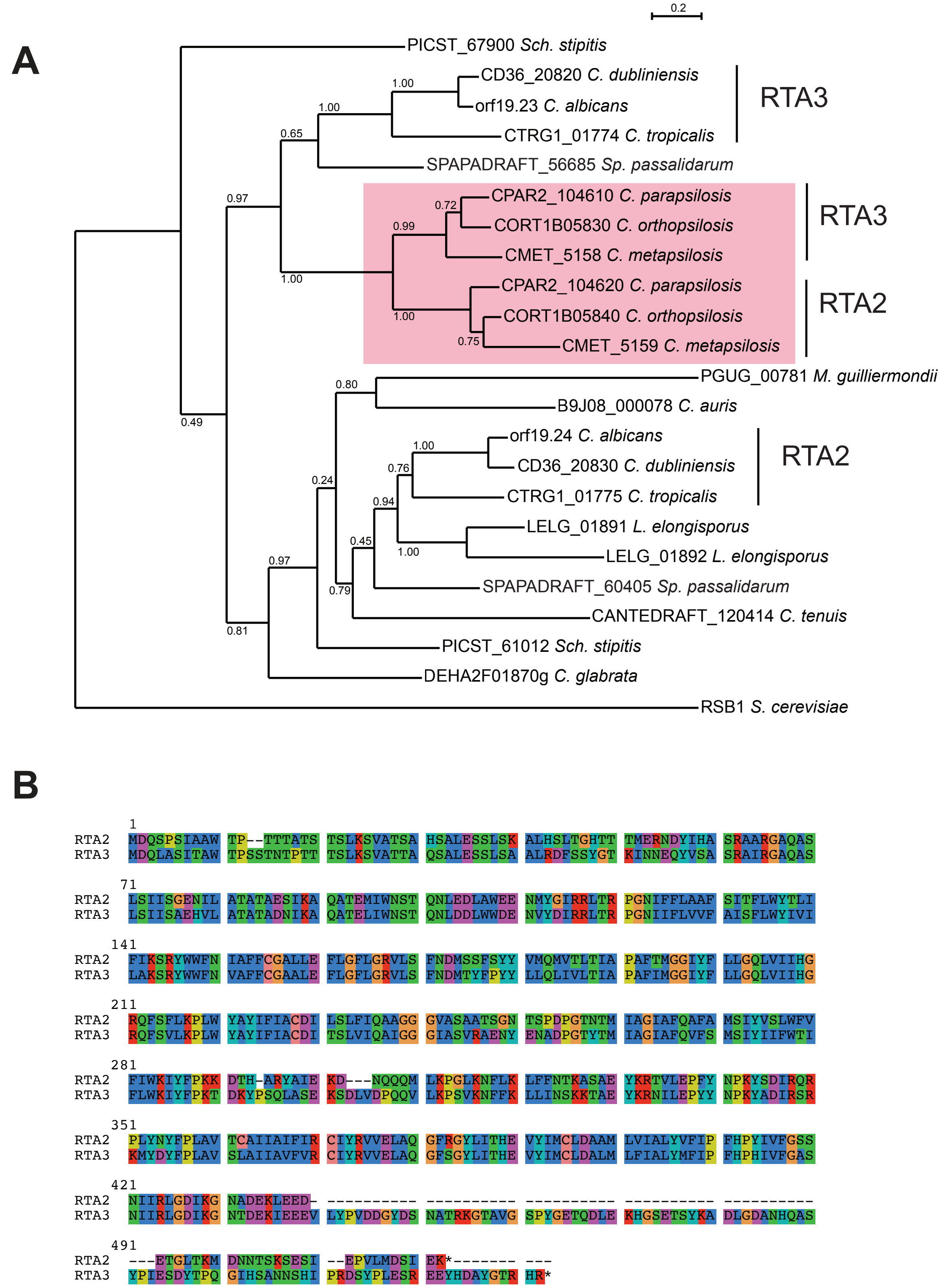


**Supplementary Figure 3. Comparison of Rta2 and Rta3 proteins.**

(A) A phylogenetic tree was generated from Muscle alignments of Rta2 and Rta3 sequences from CUG-Ser species (CGOB) using PhyML, implemented in SeaView (Gouy et al Mol Biol Evol. 2010 Feb;27(2):221-4). Bootstrap values are shown.The pink box highlights the Rta2 and Rta3 sequences from the *Candida parapsilosis* complex. The gene names are taken from the Candida Gene Order Browser (http://cgob.ucd.ie).

(B) Alignment of *C. parapsilosis* Rta2 and Rta3 generated using Muscle implemented in SeaView.
