# Supplementary material for: Systematic Analysis of Copy Number Variations in the Pathogenic Yeast Candida parapsilosis Identifies a Gene Amplification in RTA3 That is Associated with Drug Resistance
Source: mBio. 2022 Sep 19;13(5):e01777-22. doi: 10.1128/mbio.01777-22 (PMC9600344; doi:10.1128/mbio.01777-22)
Supplement: FIG S4 [file mbio.01777-22-s0007.pdf]

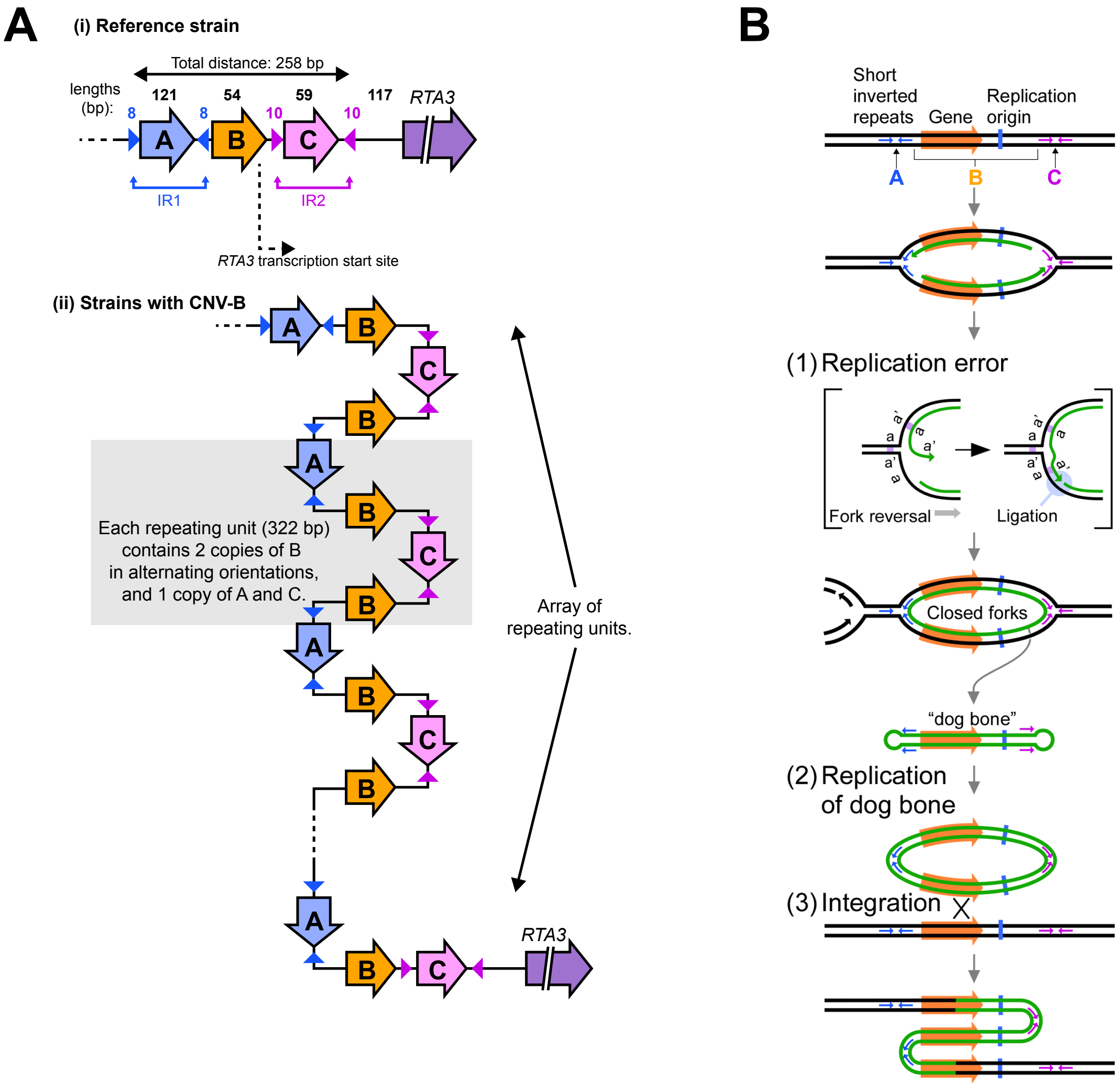

Supplementary Figure S4. Structure of CNV-B

A. (i) CNV-B consists of a central region (B) flanked by two regions (A and C) bounded by inverted repeat pairs (inward-facing triangles). The CNV occurs upstream of the *RTA3* coding sequence. (ii) CNV-B resolves as a repeat array of regions ABC interspersed with inverted copies of region B.

B. The ODIRA model of complex CNV generation, adapted from Brewer et al 2015 (PLoS Genet 11:e1005699) under the terms of the Creative Commons Attribution License. The topmost diagram has been labelled to demonstrate relationship to the observed CNV in (A).

C. (Overleaf) Replication profile of strain MSK802 mapped to the *C. parapsilosis* reference genome. Relative DNA copy number, as a proxy for replication time, is on the Y-axis, where high values denote earlier replication. The region containing RTA3 on chromosome 1 is denoted by a red bar.

C. MSKCC802

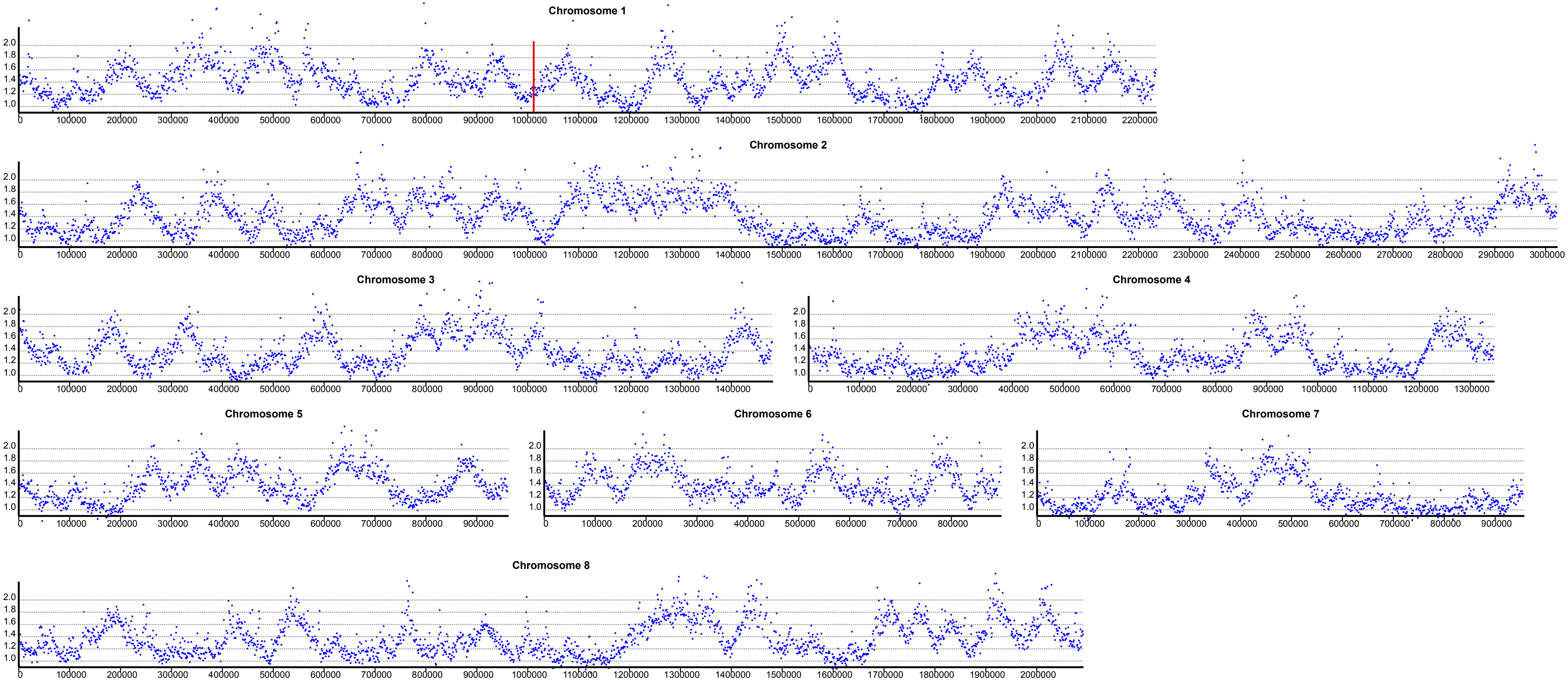
Chromosome 1
Chromosome 2
Chromosome 3
Chromosome 4
Chromosome 5
Chromosome 6
Chromosome 7
Chromosome 8
