# Supplementary material for: Systematic Analysis of Copy Number Variations in the Pathogenic Yeast Candida parapsilosis Identifies a Gene Amplification in RTA3 That is Associated with Drug Resistance
Source: mBio. 2022 Sep 19;13(5):e01777-22. doi: 10.1128/mbio.01777-22 (PMC9600344; doi:10.1128/mbio.01777-22)
Supplement: FIG S5 [file mbio.01777-22-s0008.pdf]

A

	Left breakpoints		Right breakpoints
A	acaaaatcttaaccatcaaaatagctcaagTTTTTTATTATTTTTTTTCCGTTTATTGAC	...	ACCGACTTTTTTGTTTTGTTCTTTGCAtttttcattatttttttttcttcgcctaa
B	gatttactcaaataggtggcccatgtactaAAAAAAAACCAATACAGCTCTTCTAACACT	...	TATAACTAGAGGTCACCAAAGAAAAGGaaaaacaggcattttcagctgatttcaatt
C	tttccaaatttagaccttttttcatgtgaatAAACGCCTAATGCCGAATACGTACGGAGAA	...	AATATAATTGTTGGTGTGTGCGTAGCAaaacaccaatgtcgaccagctattccatga
D	tatatgcaaaaaaaatcagcctctcctttgCATTGTGGCTGAATGAAGACCGTAAGCTAT	...	ATTGTTGGTGTGTGCGTAGCAAAACACcaatgtcgaccagctattccatgacattac
E	gtcaactagaaattagggtttcggagttaaGCAATTTTTGCTGAGATCAATTTACCCAAG	...	ATCATTCAGTTCGACTTCACTTTTTTTgcaatttttgggaacattgctgtgcctcc
F	tttgctgcttttagtgccatgtcaactagaAATTAGGGTTTCGGAGTTAAGCAATTTTTG	...	ATTTGTATTTTTCTTTACGACTCTAACcactgatggtggggcagcgcaaactacata
G	agcgcctattggagattatttgctgcttttAGTGCCATGTCAACTAGAAATTAGGGTTTC	...	CTATTGTGCAGTAATTATGCTGATTTCagtgttttcttggcgttctcatatttgtat
H	gagacaacaggttacagcaattgtagctgcGTTTGAATCCCGGTTCTATATTCGTTAAAG	...	ATACCCATTAGAAAGCAGAGAGGAGTAtcatgatgcctatggtacaagacataggta
I	ttgagtatgaggaagaggaaccaatacaaaCTAATATAGCCGTATAACGAGAACAAAATA	...	TTTGGGGAACATTGCTGTGCCTCCTCGccgctgttatcgtgcgattctgtttggcac
J	aggaagctaaagaaggaagttcgaaaaagaGAAAGGCAGATGATACAAAGTCTAAGCAAA	...	TCATGGATTCATAAAAGAAGGAAGGGTgaagctatatcaatggtttttagtgaaaag
K	caaagattaacaagattaacacaagtggcaATTACAGAAAGACGATTGGCATTGAATGAA	...	ACATGTGTGGTCTATATATGCTAGCATaaaacatctgaatcgtgtaccccaagtggt
L	atatgaaaacagctgaaagagctcatatgcCCAATAGATGGTGGGAGCGAATCAAGCTTT	...	TATTTGTATTTTTCTTTACGACTCTAAccactgatggtggggcagcgcaaactacat
M	tcggagttaagcaatttttgctgagatcaaTTTACCCAAGATGTATGTGTATATGCAAAA	...	GAAAGAATCATAAAGTGCTCATATCATtttgagctcccaaacctttcccgcgcaaca
N	tttacccaagatgtatgtgtatatgcaaaaAAAATCAGCCTCTCCTTTGCATTGTGGCTG	...	TCTCACCCAGATGGACCAGTCACCCTCaatagcagcatggactcctacaactactgc
O	acgcctaatgccgaatacgtacggagaaacTGAAAAAACAGAGGGTATCATTGGTGTAAT	...	TTTTCTTGGCGTTCTCATATTTGTATTtttctttacgactctaaccactgatggtgg
P	acgcaaaacttggatgacttgtggtgggatGAAAATGTTTATGATATTCGTCGTCTTACT	...	CAAAATTTAGAGGACTTAGCGTGGAGgaaaacatgtatggtatacgtcgtttgacac

B

CNV-1	accagcttatcaagtgaaaagttttgcaagAGGTCCAGATTCATCAGACCCACAATCATA	...	CACACCATTACCACTCCAGCAAACAAAaggactgcacgcacccctcttcattgttct
CNV-2	ctcccagaaccaaaggcttgtctcaacttcAGTTGGTTTTTTTTGTGTCAAGTAAATACG	...	CTGTCATCGACGCAACCATACACTCGAagttgactattactggggcatacacacaca
CNV-3	ataataatcgagctactcccagaaccaaagGCTTGTCTCAACTTCAGTTGTTTTTTTTGT	...	ACTATTACTGGGGCATACACACACACAcacacacacacacacacaccattaccactc
CNV-4	caatttgactcccacacagaagacatctgtACGCGGTTTATTTAAGCTAACAACATTTGC	...	TACCACTCCAGCAAACAAAAGGACTGCacgcacccctcttcattgttcttagaaaat
CNV-5	ttgattaatcgactacgaaatttgagctacCAATTTGACTCCCACACAGAAGACATCTGT	...	TGTTTTTTTCCTTCATCATCAACATCAtcaacataatcaacatcaacaaaaccattg
CNV-6	cgagcttacaaccaatatcaatccgacaatCTTGTACAACAACGTATGTAGGCCCAAATA	...	CGTGACATTTTGTGCGCATAGACACGCatacctacccacacctccacacacacatac
CNV-7	catcaatgcaattgaactcaacctccgatcACCAACTCCACCCATTTACTCAAGTAATCA	...	AAAACAACAACATACGAAGCATTAAATtaagctactcgatactatacgaattgtgag
CNV-8	tttttggtccaccaaaaaacatttgatcacCACACAAACCATTGATAACATTATCAGCGC	...	CACACACACACACACACACACACCATTaccactccagcaaacaaaaggactgcacgc

Supplementary Figure S5. Repeat sequences at (A) *RTA3* and (B) *ARR3* amplifications. Diagram showing the sequences at breakpoints for the 16 *RTA3* CNVs (A), and eight *ARR3* CNVs (B). Breakpoints are demarcated by a switch from lowercase (outside the CNV) to uppercase (covered by the CNV) and vice-versa. Text highlighted in pink shows similarity between start- and end-points
