# Supplementary material for: Systematic Analysis of Copy Number Variations in the Pathogenic Yeast Candida parapsilosis Identifies a Gene Amplification in RTA3 That is Associated with Drug Resistance
Source: mBio. 2022 Sep 19;13(5):e01777-22. doi: 10.1128/mbio.01777-22 (PMC9600344; doi:10.1128/mbio.01777-22)
Supplement: TABLE S3 [file mbio.01777-22-s0003.docx]

Supplementary Table S3. List of Primers

| **Primer:** | **Purpose:** | **Sequence:** |
| --- | --- | --- |
| **Deletion of RTA3 (CPAR2_104610)** | | |
| RTA3_KO_Guide_TOP | Oligo designed for cloning into pCP-tRNA vector as guide RNA sequence to cleave within RTA3 | CCACTGCTGTTGGAAGCCCATAT |
| RTA3_KO_Guide_BOT | Oligo designed for cloning into pCP-tRNA vector as guide RNA sequence to cleave within RTA3 | AACATATGGGCTTCCAACAGCAG |
| RTA3_KO_Upstream_Fwd | Primer designed for amplification of ~500b upstream of RTA3 | CAGAGGGTATCATTGGTG |
| RTA3_KO_Upstream_Rev | Primer designed for amplification of ~500b upstream of RTA3, includes inserted barcode for use as overlap in fusion PCR and screening transformants via colony PCR | CCCTGAAATGAGTGGTCTCTACTGGTCCATACTACTAG |
| RTA3_KO_Downstream_Fwd | Primer designed for amplification of ~500b downstream of RTA3, includes inserted barcode for use as overlap in fusion PCR and screening transformants via colony PCR | AGAGACCACTCATTTCAGGGCATAGGTAAATCTTTGGG |
| RTA3_KO_Downstream_Rev | Primer designed for amplification of ~500b downstream of RTA3 | TAGACCACACATGTTGCG |
| RTA3_Fusion_PCR_Fwd | Primer designed for use in fusion PCR of upstream and downstream amplicons | GGAAGTATCAATGTAAGCTG |
| RTA3_Fusion_PCR_Rev | Primer designed for use in fusion PCR of upstream and downstream amplicons | CATCAGTGGTTAGAGTCG |
| RTA3_Barcode_Fwd | Primer designed for amplifcation of product specific to RTA3 knockout barcode, for use with RTA3_KO_Downstream_Rev. For use in colony PCR | TGGACCAGTTAGAGACCAC |
|  |  |  |
| **Deletion of DNF1 (CPAR2_303950)** | | |
| 303950_KO_Guide_TOP | Oligo designed for cloning into pCP-tRNA vector as guide RNA sequence to cleave within CPAR2_303950 | CCAAAACGACACCTGCTGGGTGC |
| 303950_KO_Guide_BOT | Oligo designed for cloning into pCP-tRNA vector as guide RNA sequence to cleave within CPAR2_303950 | AACGCACCCAGCAGGTGTCGTTT |
| 303950_KO_Upstream_Fwd | Primer designed for amplification of ~700b upstream of CPAR2_303950 | CCTCATTCACTCCTTCTCTG |
| 303950_KO_Upstream_Rev | Primer designed for amplification of ~700b upstream of CPAR2_303950, includes inserted barcode for use as overlap in fusion PCR and screening transformants via colony PCR | TATGCAAATTCGTGCGTGTGCTTGAACAAACTAGGGTCTG |
| 303950_KO_Downstream_Fwd | Primer desgned for amplification of ~600b downstream of CPAR2_303950, includes inserted barcode for use as overlap in fusion PCR and screening transformants via colony PCR | CACACGCACGAATTTGCATAGTTGCATCGTATCAATGAAGG |
| 303950_KO_Downstream_Rev | Primer designed for amplification of ~600b downstream of CPAR2_303950 | TCCTTCGACGCCTGTTACTG |
| 303950_Fusion_PCR_Fwd | Primer designed for use in fusion PCR of upstream and downstream amplicons | TCATTCACATACACGCACAC |
| 303950_Fusion_PCR_Rev | Primer designed for use in fusion PCR of upstream and downstream amplicons | GCTTTGTCTTTCCACAGTCC |
| 303950_Barcode_Fwd | Primer designed for amplifcation of product specific to CPAR2_303950 knockout barcode, for use with 303950_Fusion_PCR_Rev. For use in colony PCR | GTTCAAGCACACGCACGA |
|  |  |  |
| **Deletion of DNF2 (CPAR2_102700)** | | |
| 102700_KO_Guide_TOP | Oligo designed for cloning into pCP-tRNA vector as guide RNA sequence to cleave within CPAR2_102700 | CCAAATGGAACTGCAGCCAACCC |
| 102700_KO_Guide_BOT | Oligo designed for cloning into pCP-tRNA vector as guide RNA sequence to cleave within CPAR2_102700 | AAC GGG TTG GCT GCA GTT CCA TT |
| 102700_KO_Upstream_Fwd | Primer designed for amplification of ~500b upstream of CPAR2_102700 | CGCCTACTGGATCTGATTGA |
| 102700_KO_Upstream_Rev | Primer designed for amplification of ~500b upstream of CPAR2_102700, includes inserted barcode for use as overlap in fusion PCR and screening transformants via colony PCR | CTAAACAATGTCCGATCCGTGACAAACACACACACACTAC |
| 102700_KO_Downstream_Fwd | Primer designed for amplification of ~500b downstream of CPAR2_102700, includes inserted barcode for use as overlap in fusion PCR and screening transformants via colony PCR | ACGGATCGGACATTGTTTAGCCCAGCTTCTTGATGTTGAT |
| 102700_KO_Downstream_Rev | Primer designed for amplification of ~500b downstream of CPAR2_102700 | CCTCTTTCAACCACTACACC |
| 102700_Fusion_PCR_Fwd | Primer designed for use in fusion PCR of upstream and downstream amplicons | CAACAAAGGTCTCTTCTACCG |
| 102700_Fusion_PCR_Rev | Primer designed for use in fusion PCR of upstream and downstream amplicons | AGCATCAAAGCTTGGGTCA |
| 102700_Barcode_Rev | Primer designed for amplification of product specific to CPAR2_102700 knockout barcode, for use with 102700_Fusion_PCR_Fwd. For use in colony PCR | GAAGCTGGGCTAAACAATGTC |
|  |  |  |
| **RT qPCR of RTA3 and ACT1** | | |
| ACT1_Fwd | Primer used for amplification of ACT1 in qPCR | TGATGACGCACCAAGAGC |
| ACT1_Rev | Primer used for amplification of ACT1 in qPCR | GACCCATACCAACCATGATACC |
| RTA3_Fwd | Primer used for amplification of RTA3 in qPCR | CCTGTAGATGACGGGTATGAC |
| RTA3_Rev | Primer used for amplification of RTA3 in qPCR | TGCATCCCCAAGATCAGC |
